# Oxovanadium-Catalyzed Epoxidation of Methyl Oleate: Ligand Effects

**DOI:** 10.1021/acsomega.6c03410

**Published:** 2026-06-19

**Authors:** Abdellatif A. Helaly, Miljan Z. Ćorović, Antoine Dupé, Yoji Kobayashi, Abdesslem Jedidi, Bambar Davaasuren, Mostafa A. Hussien, Bandar A. Babgi, Nadia C. Mösch-Zanetti

**Affiliations:** † Department of Chemistry, Faculty of Science, King Abdulaziz University (KAU), Jeddah 21589, Saudi Arabia; ‡ Institute of Chemistry, Inorganic Chemistry, University of Graz, Schubertstrasse 1, 8010 Graz, Austria; § Center of Renewable Energy and Storage Technologies (CREST), Chemistry Program, Division of Physical Sciences and Engineering, King Abdullah University of Science and Technology (KAUST), Thuwal 23955-6900, Saudi Arabia; ∥ Department of Chemistry, Faculty of Science, Damietta University, Damietta 34517, Egypt; ⊥ Imaging and Characterization Core Lab, King Abdullah University of Science and Technology (KAUST), Thuwal 23955-6900, Saudi Arabia

## Abstract

The development of catalytic reactions based on earth-abundant first-row transition metals that use chemicals from renewable feedstocks aligns with current principles of sustainable chemistry. Here, we employ oxovanadium­(IV) salen-type complexes ([VO­(Ln)], *n* = 1–5: **1**–**5**) as catalysts for the selective epoxidation of biodiesel-derived methyl oleate, where Ln are tetradentate salen-type ligands with different diamine linkers, namely ethylenediamine (**1**), 1,3-diaminopropane (**2** and **5**), diaminomaleonitrile (**3**), and 1,2-diaminocyclohexane (**4**). The 1,3-diaminopropane system was examined with both unsubstituted (*p*-H) (**2**) and substituted (*p*-OMe) (**5**) salicylaldehyde-aromatic rings. DFT analysis revealed the influence of the ligand on the electronics of the V=O moiety, with [VO­(**L3**)] exhibiting the most electron-deficient vanadium center. Notably, this complex also proved to be the most efficient epoxidation catalyst under optimized conditions (1 mol % catalyst, 3.5 equiv oxidant-TBHP, no added solvent). UV–Vis spectroscopy monitoring of the reaction between the complexes **1–5** with excess oxidant (pseudo-first order conditions) highlighted pronounced ligand-dependent differences in reactivity. Linear kinetics were observed only for [VO­(**L2**)] and [VO­(**L5**)], both containing a 1,3-diaminopropane bridge. In contrast, compounds with saturated two-carbon bridges [VO­(**L1**)] and [VO­(**L4**)] reacted slowly with the oxidant, displaying an induction period. Finally, [VO­(**L3**)] does not react with the oxidant under the same conditions, suggesting an alternative epoxidation mechanism via a V­(IV) center in the initial stage of catalysis. These results demonstrate that vanadium salen-type complexes, although structurally similar, enable epoxidation through either the commonly proposed V­(V)-peroxide pathway or a V­(IV) Lewis-acidic center, depending on the nature of the moiety bridging the two imine nitrogens.

## Graphical abstract

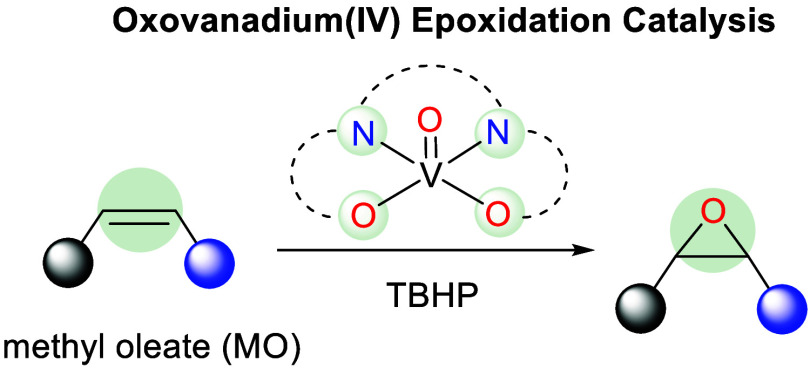

## 1 Introduction

Epoxides obtained from unsaturated fatty acids and fatty acid esters derived from renewable feedstocks, such as vegetable oils, are important intermediates in the manufacture of plasticizers, stabilizers, resins, and lubricants.^1–4^ Over the past few decades, numerous catalytic systems have been developed for the epoxidation of oleic acid and methyl oleate.^5–11^ Heterogeneous catalytic systems have attracted considerable industrial interest because of their facile catalyst recovery.^12^ Titanium-based catalysts, mesoporous transition-metal catalysts, and supported polyoxometalate systems have been reported for the epoxidation of fatty acid derivatives.^5^ Nevertheless, homogeneous catalytic systems offer the advantage of investigating catalytic activity, selectivity, and structure–activity relationships because of their well-defined active sites and mechanistic accessibility. The solvent-free homogeneous catalytic epoxidation of methyl oleate has drawn significant attention due to its green prospects and cost reduction. Many transition-metal catalysts (Mo, Re, Mn, Ru, Ti, and V) have been explored for this transformation, typically using *tert*-butylhydroperoxide (TBHP), cumene hydroperoxide (CHP), or hydrogen peroxide (H_2_O_2_) as oxidants (Scheme 1).^6–11^

**Scheme 1:**
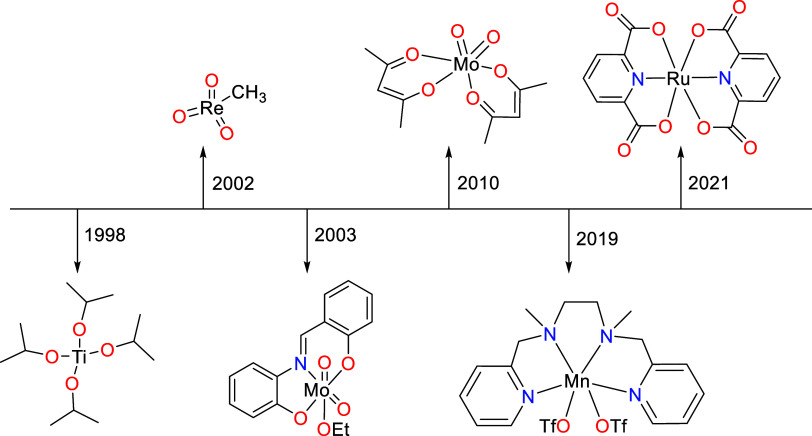
Some Transition-Metal Catalytic Systems Were Used for Epoxidation of Unsaturated Long-Chain Olefins: Titanium,^9^ Rhenium,^8^ Molybdenum,^6,7^ Manganese,^10^ and Ruthenium^11^

Molybdenum-based catalysts, such as [Mo­(O)_2_(SAP)­(EtOH)]/TBHP, where SAP is salicylidene-2-aminophenolate or [MoO_2_(acac)_2_]/TBHP, have demonstrated catalytic performance in epoxidation reactions.^6,7^ Rhenium catalysts, particularly the methyltrioxorhenium MTO–CH_2_Cl_2_/H_2_O_2_ biphasic system, exhibit catalytic efficiency under mild conditions.^8^ Titanium alkoxides, such as Ti­(OiPr)_4_, offer diastereoselective epoxidation of methyl ricinoleate/(TBHP/d-tartrate ligand).^9^ Also, manganese systems were found to be catalytic for the epoxidation of vegetable oils under mild conditions.^10^ Ruthenium catalysts, like Ru­(acac)_3_ and dipicolinic acid as a coligand, have been developed for the selective epoxidation of oleic acid using H_2_O_2_ in acetonitrile under mild conditions.^11^ Despite their demonstrated activity, most of the systems face limitations, such as the need for elevated temperatures, chlorinated solvents, inert atmospheres, acidic conditions, or costly metals.

Vanadium complexes are widely used as catalysts in different catalytic reactions, including oxidations, bond cleavages and formations,^13^ and various metathesis processes,^14^ with recent studies further demonstrating their versatility in polymerization and polymer functionalization.^15^ Oxovanadium complexes have emerged as effective catalysts for the epoxidation of olefins,^16,17^ proceeding via electrophilic V-peroxide intermediates, whose formation and oxygen atom transfer capabilities are strongly influenced by ligand electronics and steric environment.^18^ In 2016, Cecchini et al. found that oxovanadium­(IV) compounds bearing two acac-type ligands are effective catalysts for methyl oleate epoxidation. The study suggests an initial oxidation of the metal center to V­(V)-peroxide intermediates, which then transfer an oxygen atom to the substrate’s double bond.^19^ Since then, no additional studies on V-catalyzed methyl oleate epoxidation have been reported. While V-salen-type complexes have been investigated in oxygen atom transfer reactions with low molecular weight substrates,^17,20^ their reactivity toward long-chain substrates such as fatty acids and their esters remains unexplored. Therefore, we aim to examine this transformation using simple V-salen-type complexes to assess the influence of the ligand structure on catalytic activity.

## 2 Result and Discussion

### 2.1 Catalyst Preparation

Salen-type ligands were selected to investigate the effect of the ligand structure on oxovanadium­(IV)-catalyzed methyl oleate epoxidation due to their simple preparation and tunability (Scheme 2). Ligands **L1**–**L5** were synthesized from various diamine linkers to tune steric and electronic effects.^21–26^ The series includes saturated two-carbon (**L1**, **L4**) and three-carbon (**L2**, **L5**) bridges, as well as an unsaturated electron-poor backbone (**L3**). All salen-type ligands were prepared from salicylaldehyde, except for **L5**, which was prepared from 4-methoxysalicylaldehyde, to probe the influence of aromatic substitution.^22^ Oxovanadium­(IV) complexes [VO­(Ln)] (*n* = 1–5: **1**–**5**) have been prepared according to slightly modified literature-reported procedures^27,28^ and are presented in Scheme 2.

**Scheme 2:**
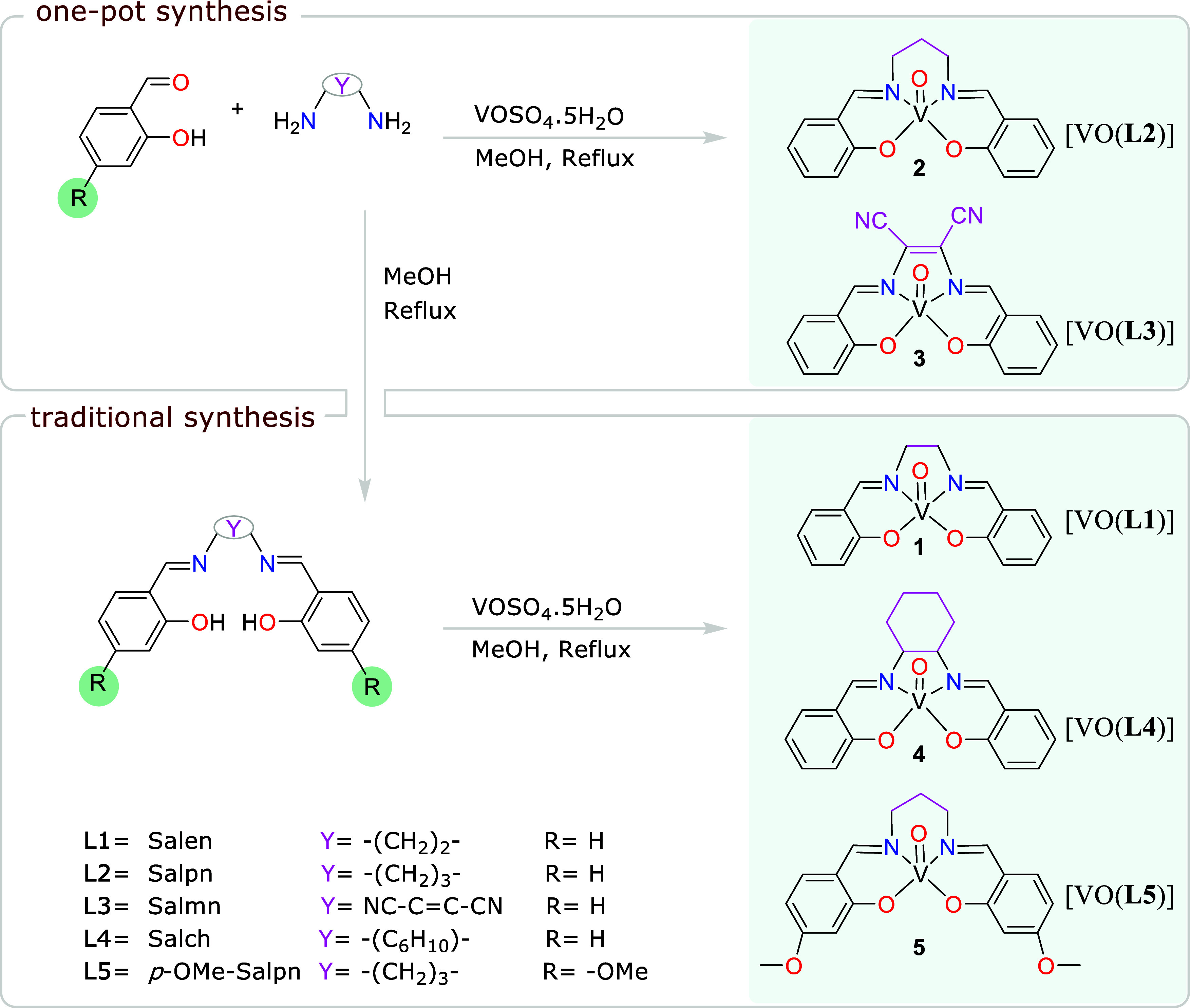
Two Synthetic Routes of Oxovanadium­(IV) Complexes[Fn s2fn1]

a
**1**, **4**, and **5** were prepared by reacting ligands **L1**, **L4**, and **L5** with VOSO_4_, whereas **2** and **3** were obtained in a one-pot synthesis (diamine + aldehyde + VOSO_4_) without prior ligand preparation.

Complexes **1**–**4** have previously been reported,^27,29–31^ in contrast to **5**, for which the synthetic procedure is found in the Supporting Information. The identity of all compounds has been confirmed by IR spectroscopy and mass spectrometry, and the data align well with those from the literature.^27,29–31^ The purity of all catalysts has been confirmed by elemental analysis. Furthermore, the molecular structure of compound **5** was determined by X-ray diffraction analysis. Suitable single crystals were obtained from a saturated DMSO solution. A molecular view of **5** is shown in Figure 1. The measurement revealed that the oxovanadium­(IV) core is coordinated by the N_2_O_2_ ligand **L5**, adopting a square-pyramidal geometry. The sixth coordination site in trans position to the oxo group is occupied by a solvent molecule (DMSO). In principle, this site is labile and can alternatively be filled with other molecules. For example, the solid-state structure of **2** reveals the presence of a bridging V–O unit, resulting in the polymeric O–V–O arrangement.^30^

**Figure 1:**
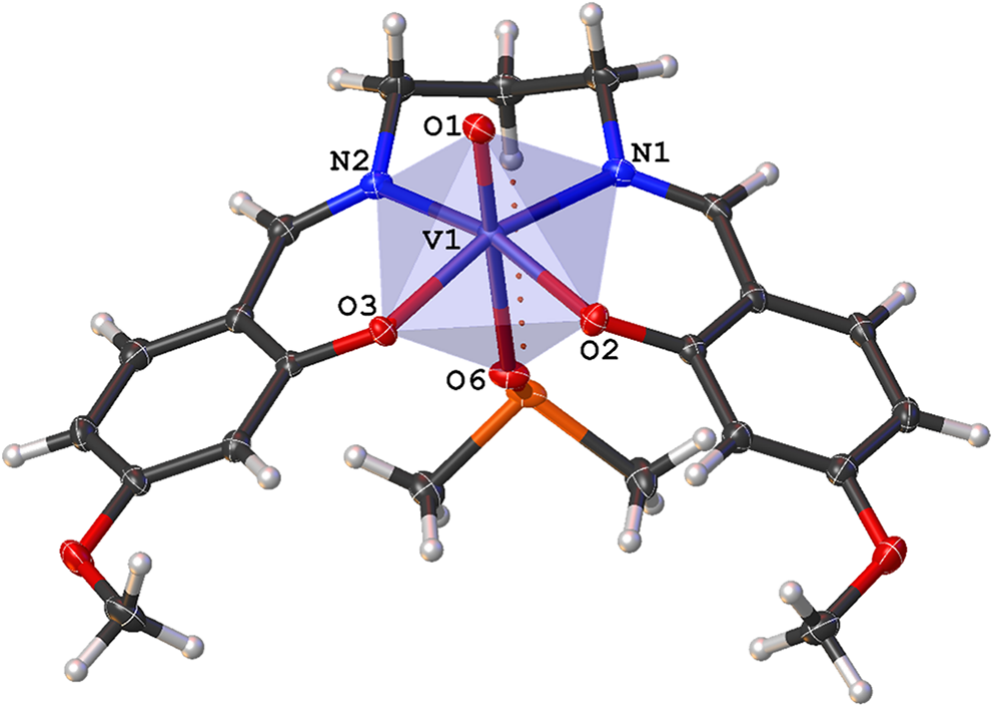
Molecular view of compound **5** [VO­(L5)­(DMSO)].

### 2.2 Optimization of the Epoxidation Reaction

The oxovanadium­(IV) catalysts **1–5** were utilized to convert methyl oleate (MO) to epoxide methyl oleate (EMO), as shown in Scheme 3. The method of one factor at a time (OFAT) was used to optimize the conditions of the epoxidation reaction using catalyst **5** by systematically varying the reaction parameters with regard to reaction time, temperature, oxidant loading (TBHP), catalyst loading, and solvent. All catalytic reactions were performed using TBHP in decane as an oxidant. The reactions were monitored by ^1^H NMR spectroscopy (Figures S1 and S2), as the starting methyl oleate and the corresponding epoxide have distinctive NMR signals.^11^ In addition, the identity of the epoxide product has been further confirmed with GC-MS measurements.

**Scheme 3:**
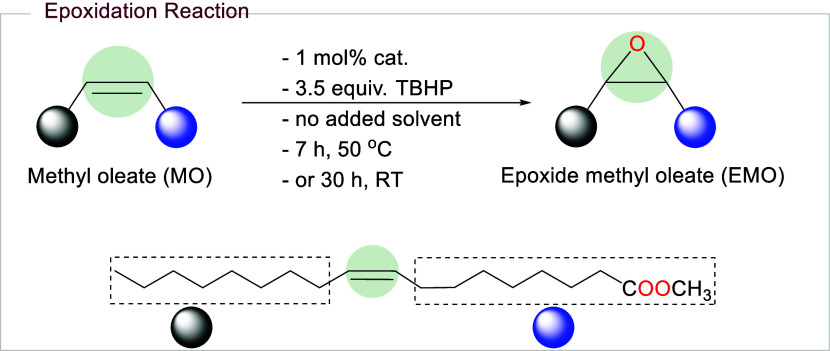
Epoxidation Reaction of Methyl Oleate under Optimized Conditions

First, the optimal reaction time was identified as 7 h, balancing complete conversion with maximum selectivity and minimizing undesired side processes.^32^ The influence of the solvent was examined (Figure 2A and Table S4): the neat reaction without added solvent, aside from the decane in TBHP, achieved the best outcome (84% conversion, 85% selectivity, 71% yield). Catalytic reactions with added solvents (toluene, decane, chloroform, and acetonitrile) were performed, ensuring identical concentrations. The catalytic performance was found to be solvent-dependent, with yields decreasing in the order toluene > decane > chloroform > acetonitrile. Coordinating solvents such as acetonitrile likely interact with the metal center, leading to reduced catalytic activity.

**Figure 2:**
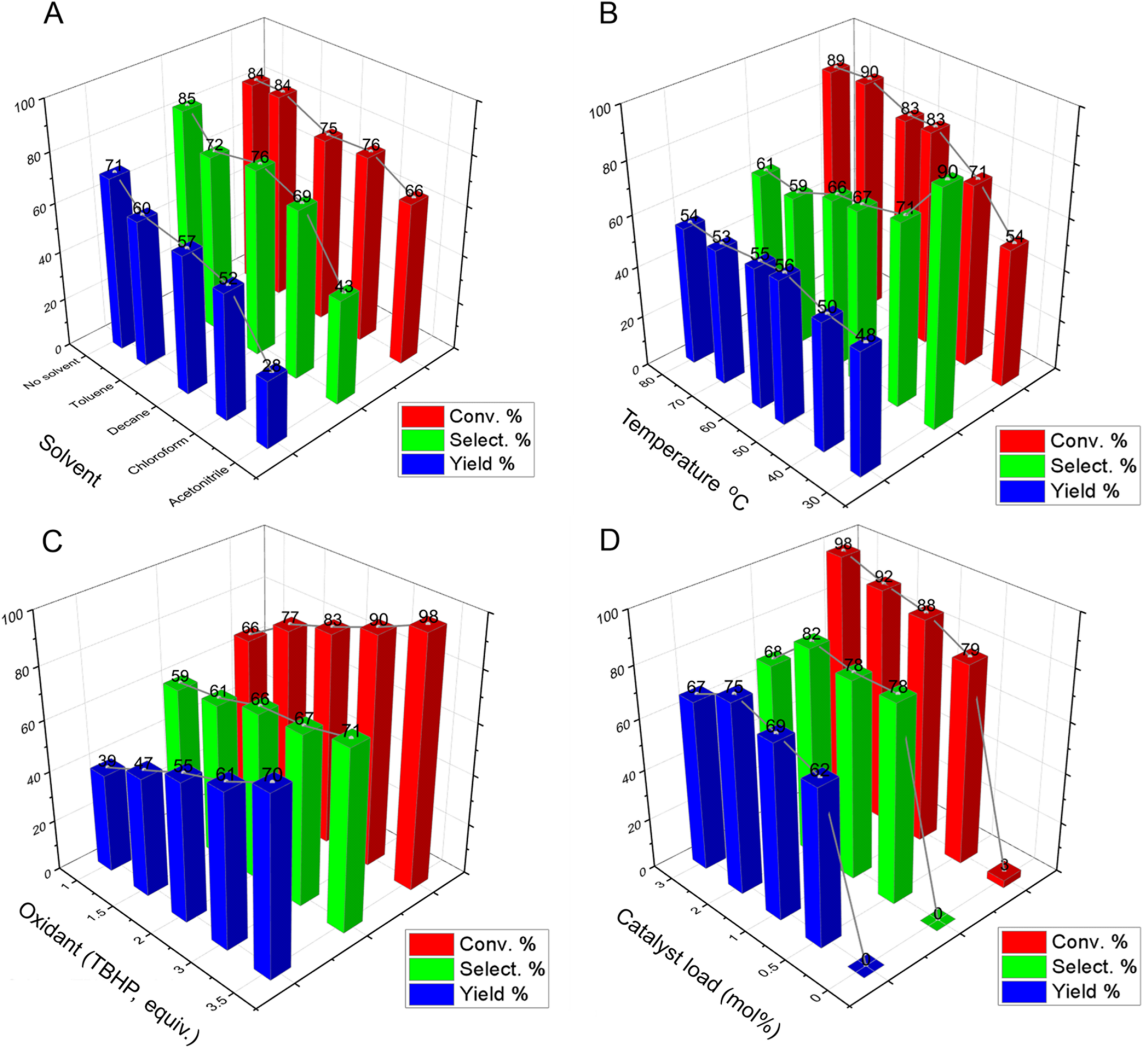
Optimization of the epoxidation conditions using catalyst **5** for all experiments. (A) Solvent screening (0.5 mmol MO, 1 mol % cat., 3.5 equiv TBHP, 50 °C, 7 h). (B) Temperature screening (0.5 mmol MO, 2 mol % cat., 2 equiv TBHP, 7 h, no added solvent, 30–80 °C). (C) Screening of TBHP loading (0.5 mmol MO, 2 mol % cat., TBHP (1.0–3.5 equiv), 7 h, no added solvent, 60 °C). (D) Catalyst load screening (0.5 mmol MO, cat. (0.5–3.0 mol %), 3.5 equiv TBHP, 7 h, no added solvent, 50 °C).

Furthermore, temperature strongly affected epoxidation of methyl oleate (Figures 2B and S4 and Table S5). At room temperature, selectivity was highest (∼90%), although conversion remained modest (54%). Conversion increased to ∼90% at 70–80 °C, but selectivity dropped to ∼60%, likely because of side reactions arising from radical formation.^32^ The yield maximized to ∼56% at 50–60 °C, defining 50 °C as the optimal balance of conversion and selectivity. Moreover, the effect of TBHP loading on methyl oleate epoxidation was examined (Figures 2C and S5 and Table S6). Increasing TBHP from 1.0 to 3.5 equiv improved conversion from 66% to 98% and selectivity from 59% to 71%, resulting in a yield increase from 39% to 70%. Catalyst loading strongly influenced epoxidation of methyl oleate (Figures 2D and S6 and Table S7). Conversion increased from 79% at 0.5 mol % to 98% at 3 mol %, while selectivity was hardly influenced (∼78–82%) up to 2 mol %, but dropped to 68% at 3 mol %. The yield peaked at 75% with 2 mol % catalyst, though 1 mol % already afforded high conversion (88%) and competitive yield (69%), identifying it as the optimal loading.

In summary, the epoxidation of methyl oleate was most efficient under the optimized conditions of 7 h at 50 °C (or 30 h at room temperature) without added solvent, using 1 mol % catalyst and 3.5 equiv of TBHP. Under optimized conditions, blank reactions without a catalyst yielded no epoxide (Table S7 and Figure S7).

### 2.3 Catalytic Performance

With the optimized conditions established, oxovanadium­(IV) complexes **1**–**5** were tested accordingly. The catalytic performances are presented in Table 1.

**Table 1:**
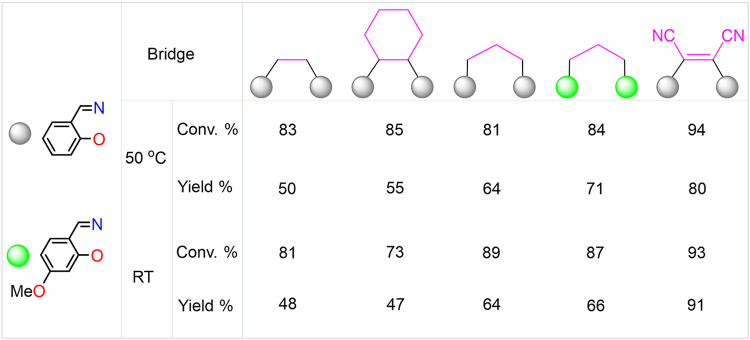
Catalytic Performance of Catalysts **1–5** under the Optimized Conditions

Catalyst screening revealed distinct effects of the ligand environment on the epoxidation of methyl oleate (Figures S8 and S9 and Table S8). Catalysts **1** and **4**, bearing a saturated two-carbon bridge, gave modest activity, with conversions of 81–85% and yields of 47–55%, reflecting only moderate selectivity (∼60–65%). These data are lower compared to VOSO_4_, which displayed higher activity (93% conversion) but also limited selectivity (63%), resulting in yields of ∼59%. Catalysts **2** and **5**, bearing a saturated three-carbon bridge, showed improved performance, particularly at elevated temperature. Catalyst **5** reached 85% selectivity and 71% yield at 50 °C, indicating that electron-donating substitution (*p*-OMe) enhances product selectivity. The most notable result was obtained with catalyst **3**, bearing a dicyano unsaturated two-carbon bridge, which consistently afforded superior selectivity (98% at RT, 85% at 50 °C) and the highest yield (91% at RT).

These findings indicate that systems with a saturated three-carbon bridge, which are more flexible, exhibit greater catalytic performance than those with a saturated two-carbon bridge. Furthermore, electron-withdrawing groups (unsaturated dicyano bridge) offer the best catalytic performance compared to the other catalysts. Thus, the electronic effect of salen-type ligands can drive the epoxidation to a better selectivity and yield.

Our VO­(IV)-salen system is comparable to that of related systems from the literature (Table 2). Molybdenum-based systems promoted TBHP-mediated epoxidation under an inert atmosphere,^6^ whereas the [Mn­(OTf)_2_(rac-BPBP)]/H_2_O_2_ system achieved nearly quantitative conversion and epoxide yield at room temperature in acetonitrile using AcOH as an acidic additive.^10^ Furthermore, VO­(IV)-4-acyl-5-pyrazolone complexes catalyzed methyl oleate epoxidation using TBHP in chloroform or without added solvents, although an inert atmosphere was required in the latter case.^19^ Overall, while our VO­(IV)-salen system shows comparable catalytic performance to these catalysts, it additionally offers solventless and non-inert conditions.

**Table 2:** Representative Homogeneous Catalytic Systems for Oleic Acid and Methyl Oleate Epoxidation

substrate	oxidant	catalyst	conditions	solvent	conv., yield (%)	ref
oleic acid	TBHP	[Mo(O)_2_(SAP)(EtOH)]	80 °C, 4.3 h, Ar, 0.5 mol % cat.	chlorobenzene	96, 84	^6^
methyl oleate	H_2_O_2_/AcOH	[Mn(OTf)_2_(rac-BPBP)]	RT, 1.25 h, 3 mol % cat.	acetonitrile	99, 99	^10^
methyl oleate	TBHP (decane)	VO(IV)-4-acyl-5-pyrazolone	70 °C, 10 h, N_2_, 2 mol % cat.	no added solvent	91, 91	^19^
methyl oleate	TBHP (aq)	VO(IV)-4-acyl-5-pyrazolone	50 °C, 6 h, 2 mol % cat.	chloroform	96, 80	^19^
methyl oleate	TBHP (decane)	VO(IV)-salen-type	50 °C, 7 h, 1 mol % cat.	no added solvent	94, 80	this work
methyl oleate	TBHP (decane)	VO(IV)-salen-type	RT, 30 h, 1 mol % cat.	no added solvent	93, 91	this work

### 2.4 Epoxidation Mechanism Survey

The generally accepted early transition-metal-catalyzed epoxidation cycle includes three main steps: (I) catalyst activation, (II) oxygen atom transfer to alkene, and (III) catalyst regeneration.^33^ On the basis of kinetic, spectroscopic, and theoretical studies, epoxidation catalysis using vanadium catalysts in the presence of O_2_, H_2_O_2_, or organic peroxides has been proposed to proceed through three general mechanistic pathways: (I) a Sharpless-type oxygen transfer, (II) a Mimoun-type insertion, and (III) a biradical pathway.^33^ To gain further insight into the reaction pathway of the system, radical trapping experiments were performed using 2,6-di*tert*-butyl-4-methylphenol (BHT) as a radical scavenger. In the presence of BHT (1 equiv to methyl oleate), the catalytic system exhibited lower methyl oleate conversion and epoxide yield (Figure S16). A strong inhibitory effect was observed for catalyst **5**, for which conversion decreased for 63% (from 84% to 31%) under identical conditions (Figures S12 and S13). A significantly lower effect was observed for catalyst **3** with a decrease of 30% (from 91% to 64%) after 7 h (Figures S14 and S15). For both, despite the lower catalytic activity, higher epoxide selectivity was maintained in the presence of BHT, suggesting suppression of radical-mediated pathways. These results suggest that the metal center may facilitate epoxidation either via a radical pathway, likely involving oxidation to V­(V), or via a nonradical pathway, likely maintaining the V­(IV) oxidation state (see below).^13^

Most often, the active oxidant in V-catalyzed epoxidations is a high-valent oxovanadium­(V) species,^13^ which could be reached via the reaction between the oxidant and oxovanadium­(IV) complexes. Such species are capable of oxygen atom transfer to the C=C bond of the substrate, thereby affording the corresponding epoxide.^34^ Here, the oxidation of catalysts **1**–**5** with TBHP in chloroform in the absence of olefin was investigated by UV–Vis spectroscopy at room temperature. Varying the excess of TBHP allowed for the determination of the pseudo-first-order rate constant (*k*_obs_) and the oxidation reaction rate (*k*). The absorbance ratio of the new species (*At*/*At*_0_) vs time for the oxidation reaction of **1–5** (Figure 3A) showed that **2** and **5** follow a pseudo-first-order kinetics, whereas **1** and **4** show an induction period preventing the extraction of kinetic parameters. Surprisingly, no absorbance changes upon oxidant addition to catalyst **3** were detected under the same conditions (30 min).

**Figure 3:**
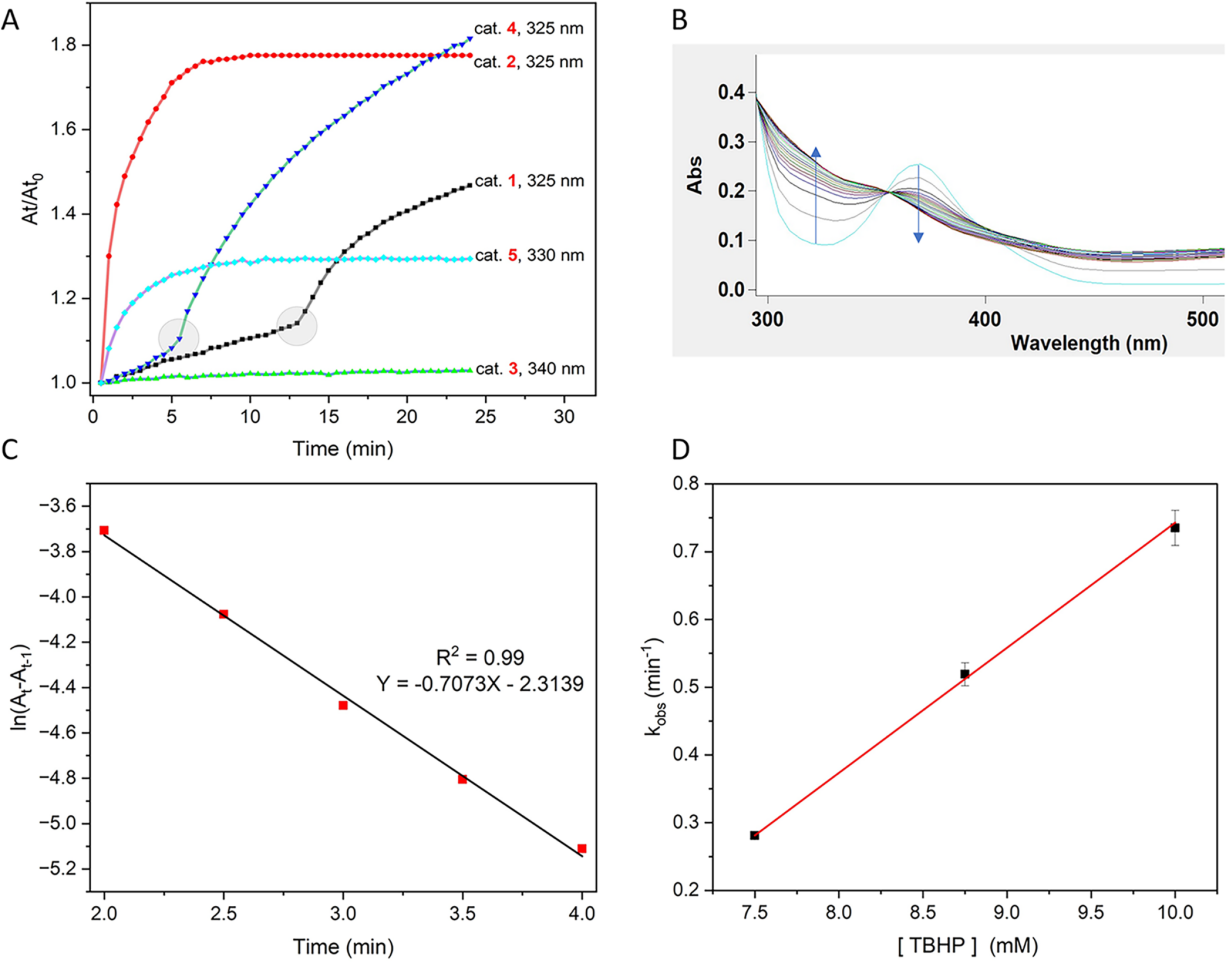
(A) Absorbance ratio of *At*/*At*_0_ vs time for the oxidation reaction of **1–5** (0.05 mM/CHCl_3_ + 200 equiv of TBHP). (B) UV–Vis spectra were recorded every 30 min during the reaction of **2** (0.05 mM in CHCl_3_) with 200 equiv TBHP; sequential spectra over time are shown in different colors. (C) Guggenheim linearization for **2** in CHCl_3_ (0.05 mM + 200 equiv TBHP). (D) Determination of the oxidation reaction rate k for complex **2** in CHCl_3_. Each measurement was repeated three times.

While the cyano-substituted complex **3** exhibits the best catalytic performance of our tested complexes, it surprisingly does not react with the oxidant for 30 min at room temperature. To further support the presence of V­(IV) species in the reaction mixture, EPR spectra of a solution containing **3** were recorded at room temperature (Figure S17) before and after addition of the oxidant (1 and 24 h). After 1 h, signals arising from the paramagnetic V­(IV) center are still present, while after 24 h, no signals are detectable. This is consistent with the UV–Vis data in the early stage of the reaction, revealing a remarkably high stability of the V­(IV) center in **3**. Longer reaction time leads to oxidation to V­(V). This suggests that catalysis in the early stage proceeds via the V­(IV) center, while late-stage catalysis might also involve V­(V).

The observed catalytic activity in oxygen atom transfer reactions could therefore be attributed to the ability of the V­(IV) center to facilitate redox processes between the substrate (MO) and the oxidant (TBHP), although it does not reach its highest oxidation state. As the most electron-poor vanadium center in this series (as confirmed by the DFT investigation shown below), this species is likely more difficult to be oxidized than the other, more electron-rich vanadium centers of complexes studied here. Although no epoxidation mechanism relying solely on vanadium­(IV) has been reported, a study of the cyclohexene epoxidation with [VO­(acac)_2_] and TBHP revealed that, in this system, several active species catalyze the reaction. These include V­(IV)-peroxide complexes alongside the more commonly proposed V­(V) species.^34^

For the electron-poor salen-type complex **3**, the most plausible epoxidation mechanism proceeds without a change of the oxidation state, at least in the early stage, similar to those suggested by the Speybroeck group.^34^ The oxovanadium­(IV) core activates *tert*-butylhydroperoxide through coordination, enabling the direct transfer of the terminal oxygen atom to the incoming olefin. Subsequent release of the epoxide is followed by protonation of one phenolate donor, generating a transient alkoxide intermediate. This species rapidly reorganizes to regenerate the starting complex. The proposed mechanism is shown in Scheme 4.

**Scheme 4:**
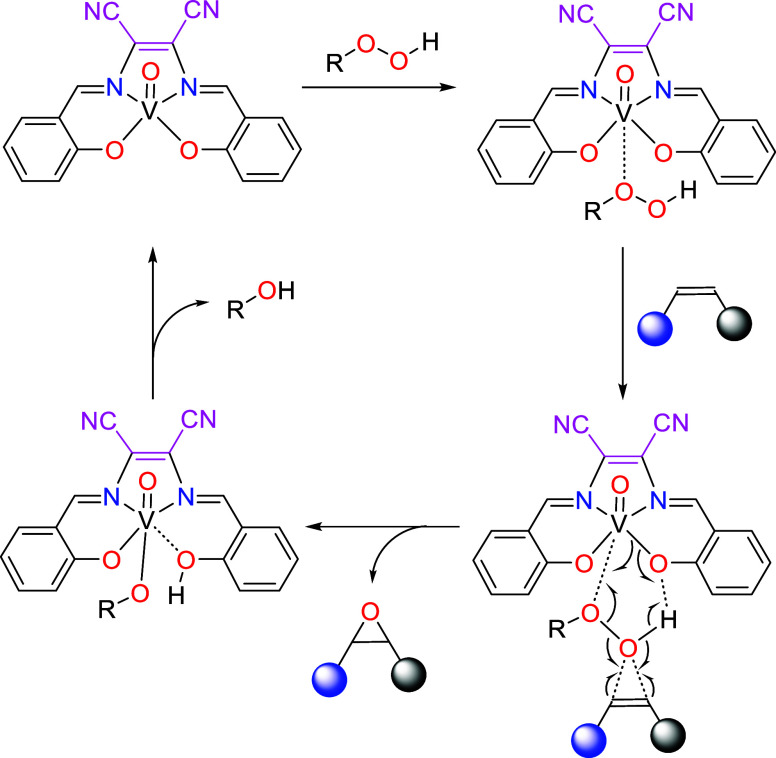
Proposed Mechanism for Methyl Oleate Epoxidation with Complex **3**

The high selectivity observed for complex **3** at room temperature supports the proposed mechanism. Oxidation of V­(IV) to V­(V) is typically associated with the formation of radical intermediates, and such species often promote side reactions reflected in byproducts. The reason that this complex does not undergo oxidation is probably due to the electron-withdrawing character of the cyano groups in the ligand backbone and the rigidity of the unsaturated salen-type framework.

The kinetic profiles of the oxidation reactions catalyzed by complexes **2** and **5** allow evaluation of the effect of the methoxy group in the ligand backbone. UV–vis spectra of the reaction of catalysts **2** and **5** with TBHP as shown in Figures 3B and S18A reveal isosbestic points, and the applied Guggenheim linearization (Figures 3C and S18B) illustrates the pseudo-first-order reactions.^35,36^ The calculated oxidation reaction rates *k* (eq S1 and Figures 3D and S18C) and the Gibbs free energies (Δ*G* ^‡^) (eq S2 and Figure S18D) were found to be *k* = 0.1816 min^–1^ and Δ*G* ^‡^ = 93.10 kJ/mol for **2** and *k* = 0.0344 min^–1^ and Δ*G* ^‡^ = 88.91 kJ/mol for **5**. Our observation that electron-donating para-substitution (**2** with *p*-H vs **5** with *p*-OMe) slows the oxidation step (formation of the V–peroxide active species) by 5.3 times is consistent with previous mechanistic and computational studies on vanadium salen-type epoxidation catalysts.^37–39^ Therefore, the measured kinetic parameters reflect only the differences in the catalyst activation step rather than substantial changes in the catalytic cycle. Although catalyst **2** exhibits a 5.3-fold higher rate constant than catalyst **5**, the relatively small difference in Δ*G* ^‡^ suggests similar reaction thermodynamics. Accordingly, both catalysts achieve comparable conversions after 5 h (Figures S10 and S11), indicating that electron-donating para-substitution has little influence on overall conversion and epoxide yield.

### 2.5 Computational Studies

Catalyst screening established the performance order **3** > **5** > **2** > **4** > **1** under optimized conditions. DFT-optimized structures for catalysts **1**–**5** display the expected terminal V=O motif, where the metal-oxo polarization Δ*q* ≡ *q*(V) – |*q*(O)| was extracted from NBO charges (Figures S19 and S20 and Table S9).^38^ The independent calculations using Becke, 3-parameter, Lee–Yang–Parr hybrid functional (B3LYP) preserved the similar trend to the catalytic performance, underscoring the use of Δ*q* for this set. Plotting catalytic activity against Δ*q* reveals various relationships: yield vs Δ*q* gives *R* ^2^ = 0.96 (RT) and 0.91 (50 °C); selectivity vs Δ*q* gives *R* ^2^ = 0.91 (RT) and 0.80 (50 °C); and in contrast, conversion vs Δ*q* gives *R* ^2^ = 0.79 (RT) and *R* ^2^ = 0.42 (50 °C),^40^ as shown in Figures 4A,B, S21, and S22. These results indicate that greater V=O polarization (larger Δ*q*) correlates with higher yield and selectivity. A simple mechanistic picture follows: increasing Δ*q* renders the V=O unit more electrophilic, which can help in the oxygen-transfer step (OAT from oxygen source TBHP to the double bond of substrate).^18^ Overall, the descriptor Δ*q* suggests higher catalytic activity for catalysts (**3**, **2**, **5**) with Δ*q* ≳ 0.49, whereas those with Δ*q* ≲ 0.49 (**4**, **1**) have comparatively lower catalytic activity.

**Figure 4:**
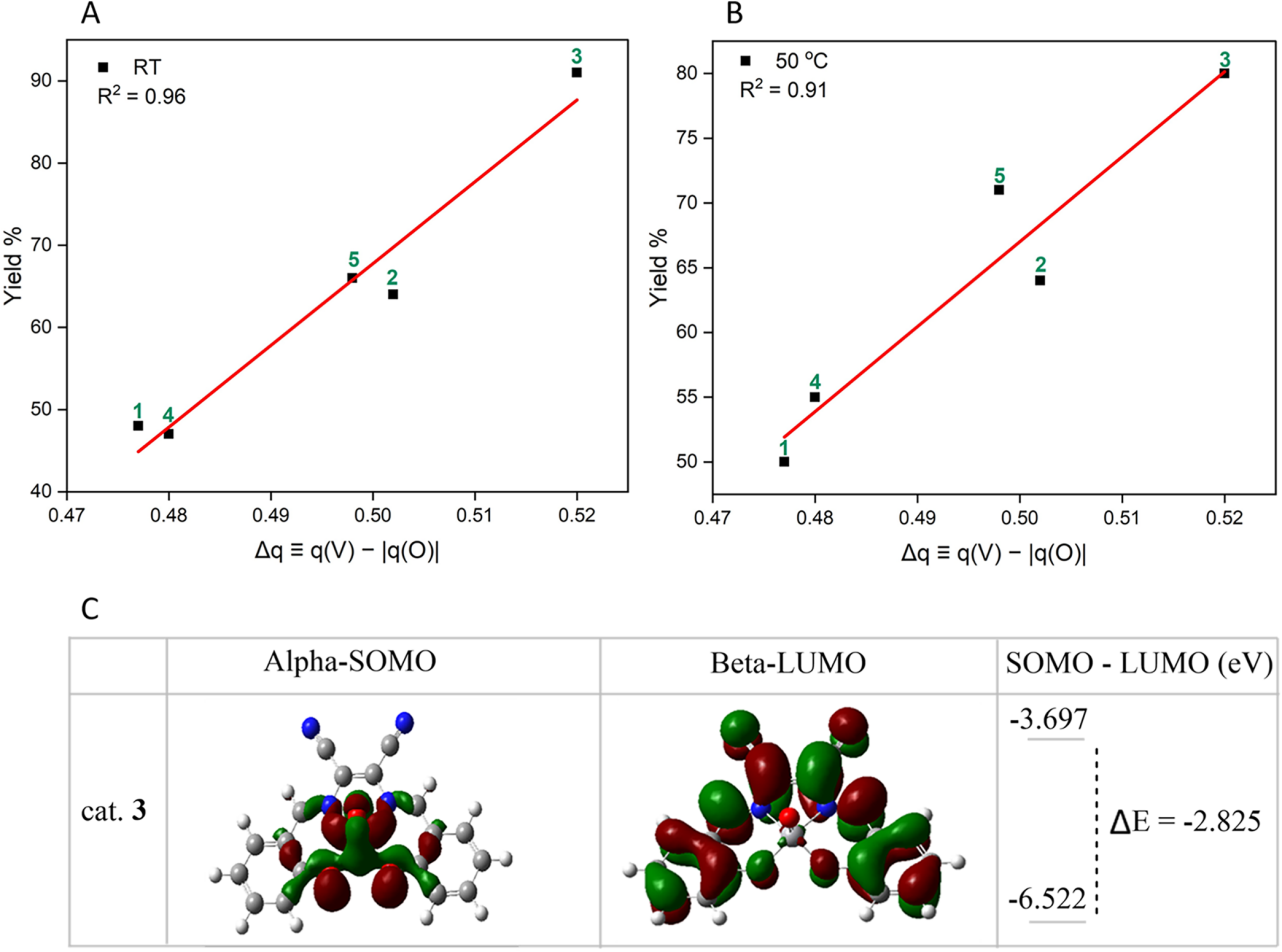
Correlation of epoxide yield (EMO) with V=O polarization Δ*q* obtained from NBO charges using B3LYP/(def2TZVP/SDD) at room temperature (A) and 50 °C (B). (C) Frontier molecular orbitals (α-SOMO and β-LUMO) of catalysts **3**.

Catalysts with larger Δq values (notably **3**) exhibit better oxygen atom transfer capability once activated. Catalyst **3** shows the smallest |Δ*E*| (2.83 eV) as shown in (Figures 4C and S23). The compressed energy gap facilitates internal charge reorganization and promotes oxygen atom transfer. In contrast, complexes **2** and **5** possess relatively higher |Δ*E*| energies (3.644 and 3.786 eV). Catalysts **1** and **4** possess moderate |Δ*E*| energies (3.528, 3.586). The correlation between small |Δ*E*|, high Δ*q*, and catalytic performance underscores the role of V=O polarization in tuning vanadium-mediated epoxidation.

## 3 Conclusion

In this work, the influence of the ligand backbone on the catalytic performance of oxovanadium­(IV) complexes was systematically investigated using the epoxidation of methyl oleate as a model reaction. A series of salen-type ligands was employed, and the study included catalyst synthesis, optimization of reaction conditions, and mechanistic investigations aimed at elucidating structure–activity relationships. DFT-optimized structures of complexes **1**–**5** revealed distinct degrees of polarization of the V=O moiety, providing a molecular basis for the observed reactivity trends. Among the complexes studied, catalyst **3** with an electron-poor, unsaturated bridge between the two imine nitrogen atoms exhibited the highest catalytic efficiency. Notably, this complex was found to be stable toward oxidation in the early stage of catalysis, indicating that epoxidation can proceed via a mechanism without changing the vanadium­(IV) oxidation state. This finding challenges the prevailing view that vanadium-catalyzed epoxidations necessarily involve V­(V)-peroxide intermediates. In contrast, complexes bearing saturated two- and three-carbon bridges displayed lower catalytic activity and reacted readily with the oxidant, consistent with their more electron-rich vanadium centers. Complexes **2** and **5**, containing three-carbon bridges, followed pseudo-first-order kinetics, most likely due to their robust coordination environment, which maintains an open coordination site for oxidant access. Overall, these results highlight the decisive role of ligand backbone electronics and rigidity in governing both the activity and the mechanistic pathway of oxovanadium­(IV) catalysts. Electron-poor vanadium complexes with robust coordination emerge as promising platforms for the development of efficient V-catalyzed epoxidation systems.

## 4 Experimental Section

Five oxovanadium­(IV) salen-type complexes featuring various diamine linkers and salicylaldehyde-derived frameworks were synthesized and evaluated as catalysts for the epoxidation of methyl oleate. The salen-type ligands were characterized by FTIR, ^1^H NMR, and ^13^C NMR spectroscopy,^21–26^ whereas the oxovanadium­(IV) complexes were characterized by FTIR spectroscopy, mass spectrometry, CHNS elemental analysis, and single-crystal X-ray diffraction for complex **5**.^27–31^ Catalytic epoxidation reactions were carried out using methyl oleate as the substrate and TBHP (5.64 M in decane) as the oxidant without the addition of an external reaction solvent. Reaction parameters were optimized using a one-factor-at-a-time (OFAT) approach. Substrate conversion and product selectivity were determined by ^1^H NMR spectroscopy.^11,41,42^ Catalyst activation in the presence of TBHP was monitored by UV–Vis spectroscopy for all complexes, while kinetic studies under pseudo-first-order conditions were performed for complexes **2** and **5**.^35–39^ Density functional theory (DFT) calculations were performed at the B3LYP level of theory, and NBO charge analysis was used to derive the electronic descriptor Δ*q* for correlation with catalytic performance.^38,40,43–46^ Full experimental procedures, characterization data, catalytic and kinetic studies, computational details, and crystallographic information are provided in the Supporting Information.

## Supplementary Material



## Notes

Full experimental procedures, computational study, and crystallographic data. (PDF)
