# Supplementary material for: Oxovanadium-Catalyzed Epoxidation of Methyl Oleate: Ligand Effects
Source: ACS Omega. 2026 Jun 19;11(25):38085–94. doi: 10.1021/acsomega.6c03410 (PMC13325083; doi:10.1021/acsomega.6c03410)
Supplement: Supplementary file 1 [file ao6c03410_si_001.pdf]

Supporting Information

Oxovanadium-Catalyzed Epoxidation of Methyl Oleate: Ligand Effects

Abdellatif A. Helaly,[†,#,§,‖] Miljan Z. Ćorović,[#] Antoine Dupé,[#] Yoji Kobayashi,[§] Abdesslem Jedidi,[†] Bambar Davaasuren,[⊥] Mostafa A. Hussien,[†] Bandar A. Babgi,[†] Nadia C. Mösch-Zanetti[#,*]

[†] Department of Chemistry, Faculty of Science, King Abdulaziz University (KAU), Jeddah 21589, Saudi Arabia
[#] Institute of Chemistry, Inorganic Chemistry, University of Graz, Schubertstrasse 1, 8010 Graz, Austria
[§] Center of Renewable Energy and Storage Technologies (CREST), Chemistry Program, Division of Physical Sciences and Engineering, King Abdullah University of Science and Technology (KAUST), Thuwal, 23955-6900, Saudi Arabia
[‖] Department of Chemistry, Faculty of Science, Damietta University, Damietta 34517, Egypt
[⊥] Imaging and Characterization Core Lab, King Abdullah University of Science and Technology (KAUST), Thuwal, 23955-6900, Saudi Arabia

*Corresponding Author: nadia.moesch@uni-graz.at

Contents

1. General Considerations S2
2. Materials S2
3. Synthesis of Ligands S3
4. Synthesis of Complexes S4
5. Catalytic Study S6
6. Catalyst Screening S16
7. Mechanistic Study S18
8. Kinetic Study S21
9. Computational Study S23
10. Crystal Structure S28

1. General Considerations

All experiments were performed under ambient atmosphere. ^{1}H NMR and ^{13}C NMR spectra were recorded on a Bruker Avance III 300 MHz spectrometer at room temperature. Chemical shifts δ are given in ppm. Solid state IR spectra were measured on a Bruker ALPHA ATR-FT-IR spectrometer at a resolution of 2 cm^{-1}. Elemental analyses (C, H, N, S) were performed at the Department of Inorganic Chemistry at the Graz University of Technology using a Heraeus Vario Elementar automatic analyzer. Values for elemental analyses are given as percentages. Mass spectroscopy measurements using electron impact ionization (EI-MS) were performed with an Agilent 5973 MSD with a push rod for direct sample measurement. GC-MS analyses were performed with an Agilent 7890A GC system with an Agilent 19091J-433 column coupled to a 5975C inert XL EI/CI mass selective detector (MSD). UV–Vis spectra were acquired with a Varian Cary 50 spectrophotometer equipped with a VWR thermostat. EPR spectra were recorded at room temperature on a Bruker Xenon X-band spectrometer operating at 9.43 GHz using a microwave power of 2.0 mW and a modulation frequency of 100 kHz.

2. Materials

Methyl oleate (MO), ethylenediamine, 5.64 M *tert*-butyl hydroperoxide(TBHP)/n-decane, 1,2-diaminocyclohexane, diaminomaleonitrile, 1,3-diaminopropane, salicylaldehyde, 4-methoxy salicylaldehyde, vanadyl sulphate pentahydrate ($VOSO_4 \cdot 5H_2O$) were purchased from commercial sources and used as received.

3. Synthesis of Ligands

Symmetrical salen-type ligands **L1**, **L4**, and **L5** were prepared via a condensation reaction between the corresponding salicylaldehyde derivatives and diamines in methanol under reflux conditions (**Scheme S1**), employing a 1:2 molar ratio of diamine to aldehyde.[1,2,3,4,5,6] Upon completion of the reaction, the resulting precipitates were isolated by filtration, thoroughly washed with cold methanol and subsequently dried in an oven at 60 °C (**Table S1**). **L2** and **L3** were not synthesized but formed in-situ during complex synthesis.[7]

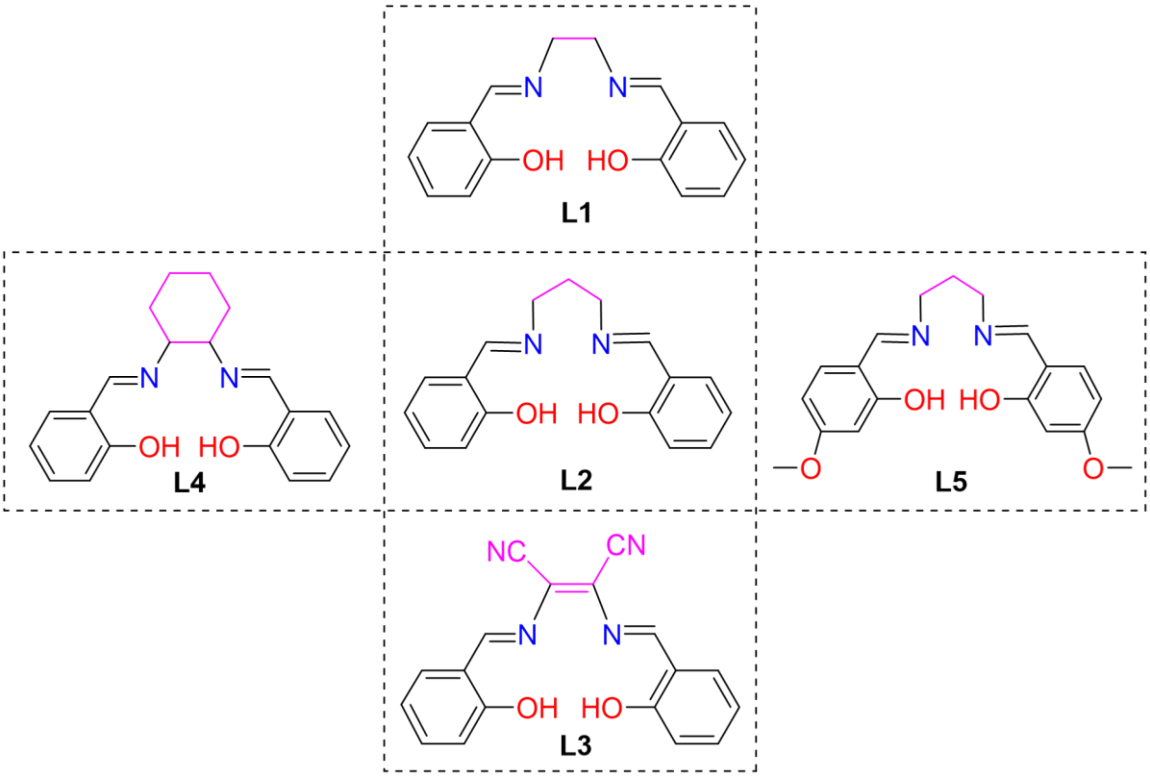

Scheme S1. The scope of salen-type ligands.

Table S1. The spectroscopic data for synthesized Ligand **L5**.

Ligand	Characterization	Ref.
**L5**	**^{1}H NMR** ($CDCl_3$, δ/ppm): 2.08 (m, 2H, –C<u>H</u>$_2$–bridge), 3.65 (t, 4H, N–C<u>H</u>$_2$–bridge), 3.8 (s, 6H, –OC<u>H</u>$_3$), 6.4-7.1 (6H, Ar–<u>H</u>), 8.2 (s, 2H, <u>H</u>–C=N), 13.90 (s, 2H, O<u>H</u>).<br>**^{13}C NMR** ($CDCl_3$, δ/ppm): 31.7 (–<u>C</u>H$_2$–bridge), 55.3–55.4 (N–<u>C</u>H$_2$ and O–<u>C</u>H$_3$ groups), 101.2-132.5 (Ar-<u>C</u>), 163.6 (–<u>C</u>=N), 164.4 (<u>C</u>-OH), 165.5 (<u>C</u>–OCH$_3$).	[5,8]

L5 was synthesised from 1,3-diaminopropane and 4-methoxysalicyaldehyde.

4. Synthesis of Complexes

Complexes **1**, **2**, **3** and **4** were prepared according to slightly modified literature procedures (**Table S2**).[9,10,7,11]

[VO(L1)] (1): $VOSO_4{\cdot}5H_2O$ (0.253 g, 1 mmol) and **L1** (0.268 g, 1 mmol) were mixed in methanol (150 ml) and heated to refluxing temperature for 4 h, the precipitate was filtered off, washed with methanol and dried at 70 °C.[9]

[VO(L2)] (2): $VOSO_4{\cdot}5H_2O$ (1.012 g, 4 mmol) and salicylaldehyde (0.85 ml, 8 mmol) were separately dissolved in methanol (150 ml) and then mixed to yield a clear green solution. A methanolic solution of 1,3-propanediamine (0.33 ml, 4 mmol) was added dropwise, resulting in the formation of yellowish-orange precipitate. The mixture was stirred reflux for 4 hours, after which the formed precipitate was filtered, washed thoroughly with methanol, and dried under vacuum.[10,7]

[VO(L3)] (3): $VOSO_4{\cdot}5H_2O$ (1.012 g, 4 mmol) and salicylaldehyde (0.85 ml, 8 mmol) methanolic solutions were mixed to give a clear green solution. The solution of diaminomaleonitrile (0.432 g, 4 mmol) was added dropwise to give an intensive red solution which was stirred under reflux for 4 h. The reddish-brown precipitate was filtered off, washed with methanol, and dried under vacuum.[7]

[VO (L4)] (4): $VOSO_4{\cdot}5H_2O$ (0.253 g, 1 mmol) and **L4** (0.322 g, 1 mmol) methanolic solutions were mixed and stirred under reflux for 4 h. Thereafter, the precipitate was filtered off, washed with methanol and dried at 70 °C.[11]

[VO (L5)] (5):

Method A): $VOSO_4{\cdot}5H_2O$ (0.352 g, 139 mmol) and **L5** (0.476g, 139 mmol) methanolic solutions were combined and stirred under reflux for 4 hours. Upon cooling, a yellowish-orange precipitate formed, which was collected by filtration, washed with methanol and water, and dried in an oven at 70 °C.

Method B): $VOSO_4{\cdot}5H_2O$ (1.012 g, 4 mmol) and 4-methoxysalicyaldehyde (1.217 g, 8 mmol) were dissolved separately in methanol (150 ml) and mixed to give a clear green solution. To this mixture, a methanolic solution of 1,3-diaminopropane (0.33 ml, 4 mmol) was added dropwise, resulting in the formation of an intense yellowish-orange precipitate. The reaction mixture was stirred under reflux for 4 h, after which the precipitate was collected by filtration, washed with methanol, and dried under vacuum.

Table S2. The spectroscopic data for synthesised complexes **1-5**.

Catalyst	Characterization	Ref.
**1**	**M/Z**: 333.2.<br>**IR** ν (cm^{-1}): 1615 (C=N), 984 (V=O).<br>**Molecular formula**: $C_{16}H_{14}N_2O_3V$<br>**Elemental analysis** (C, H, N): found C 57.29; H 4.12; N 8.35 %; calculated: C 57.67; H 4.23; N 8.41 %.	9
**2**	**M/Z**: 347.2.<br>**IR** ν (cm^{-1}): 1625 (C=N), 854 (V=O).<br>**Molecular formula**: $C_{17}H_{16}N_2O_3V$<br>**Elemental analysis** (C, H, N): found C 58.53, H 4.38, N 8.03. calculated C 58.80, H 4.64, N 8.07.	10,7
**3**	**M/Z**: 381.1.<br>**IR** ν (cm^{-1}): 1605 (C=N), 988 (V=O).<br>**Molecular formula**: $C_{18}H_{10}N_4O_3V$<br>**Elemental analysis** (C, H, N): found C 56.77; H 2.40; N 14.93 %; calculated: C 56.71; H 2.64; N 14.70 %.	7
**4**	**M/Z**: 387.3.<br>**IR** ν (cm^{-1}): 1615 (C=N), 984 (V=O).<br>**Molecular formula**: $C_{20}H_{20}N_2O_3V$<br>**Elemental analysis** (C, H, N): found C 61.77; H 4.70; N 7.10 %; calculated: C 62.02; H 5.20; N 7.23 %.	11
**5**	**M/Z**: 407.3.<br>**IR** ν (cm^{-1}): 1626 (C=N), 853 (V=O).<br>**Molecular formula**: $C_{19}H_{20}N_2O_5V$<br>**Elemental analysis** (C, H, N): found C 55.45; H 4.78; N 6.74 %; calculated: C 56.03; H 4.95; N 6.88 %.	This Work

5. Catalytic Study

1. Optimization of Epoxidation Reaction

Catalytic epoxidation experiments of methyl oleate (MO) were carried out in a 4 mL glass vial under temperature-controlled conditions. In a typical experiment, the oxovanadium catalyst was mixed with *tert*-butyl hydroperoxide (TBHP) at selected molar ratios of catalyst to MO (0.5, 1.0, 2.0, or 3.0 mol%) and varying oxidant equivalents (1, 1.5, 2.0, 3.0, or 3.5 equiv.) relative to the substrate. Methyl oleate (170 µL, 0.5 mmol) was added, and the reaction mixture was heated using a temperature-controlled heating block on a hot plate with magnetic stirring to the desired temperature (30, 40, 50, 60, 70, or 80 °C). The employed parameter ranges are summarized in **Table S3**. The reactions were followed via ^{1}H NMR spectroscopy (50 µl aliquot in 0.5 mL $CDCl_3$).

Table S3. Parameters for optimizing the epoxidation reaction.

Parameter			Range
1	Reaction temperature	°C	30, 40, 50, 60, 70, and 80
2	Catalyst loading	mol%	0, 0.5, 1, 2, and 3
3	Added solvent		$CHCl_3$, ACN, toluene, decane, and without
4	Oxidant (TBHP)	equiv.	1, 1.5, 2.0, 3.0, and 3.5
6	Reaction time	hour	at rt (0, 2, 4, 6, and 7, 24, 30, 33)<br>at 50 °C (0, 2, 4, 6, and 7)

2. NMR Monitoring of Methyl Oleate Epoxidation

The progress of the catalytic epoxidation of methyl oleate (MO) was monitored using ^1H NMR spectroscopy. Reaction aliquots were taken at selected time intervals and analysed by ^1H NMR spectroscopy in $CDCl_3$ by integrating the characteristic olefinic protons (C=C) of methyl oleate at approximately 5.3 ppm (**Figure S1**), and the epoxide protons of the product, the epoxidized methyl oleate (EMO), appearing between 2.85 and 3.10 ppm (**Figure S2**).

The protons of the methoxy group of methyl oleate ($-OCH_3$), observed as a singlet at ~3.6 ppm and chemically unaltered during the reaction, served for quantifying both substrate and product integrals.[12] Conversion and selectivity were calculated using the following equations:

$$\text{Conversion }\% = 1 - \frac{(I_t / I_{OMe,\,t})}{(I_0 / I_{OMe,\,0})} * 100 \qquad \text{Selectivity }\% = \frac{(I_{p,t})}{(I_0 - I_t)} * 100$$

Where: I_0 = Substrate integral at time zero, I_t = Substrate integral at time t, $I_{OMe,0}$ = Integral of methoxy protons at time zero, $I_{OMe,t}$ = Integral of methoxy protons at time t, $I_{p,t}$ = Integral of product epoxide protons at time t.

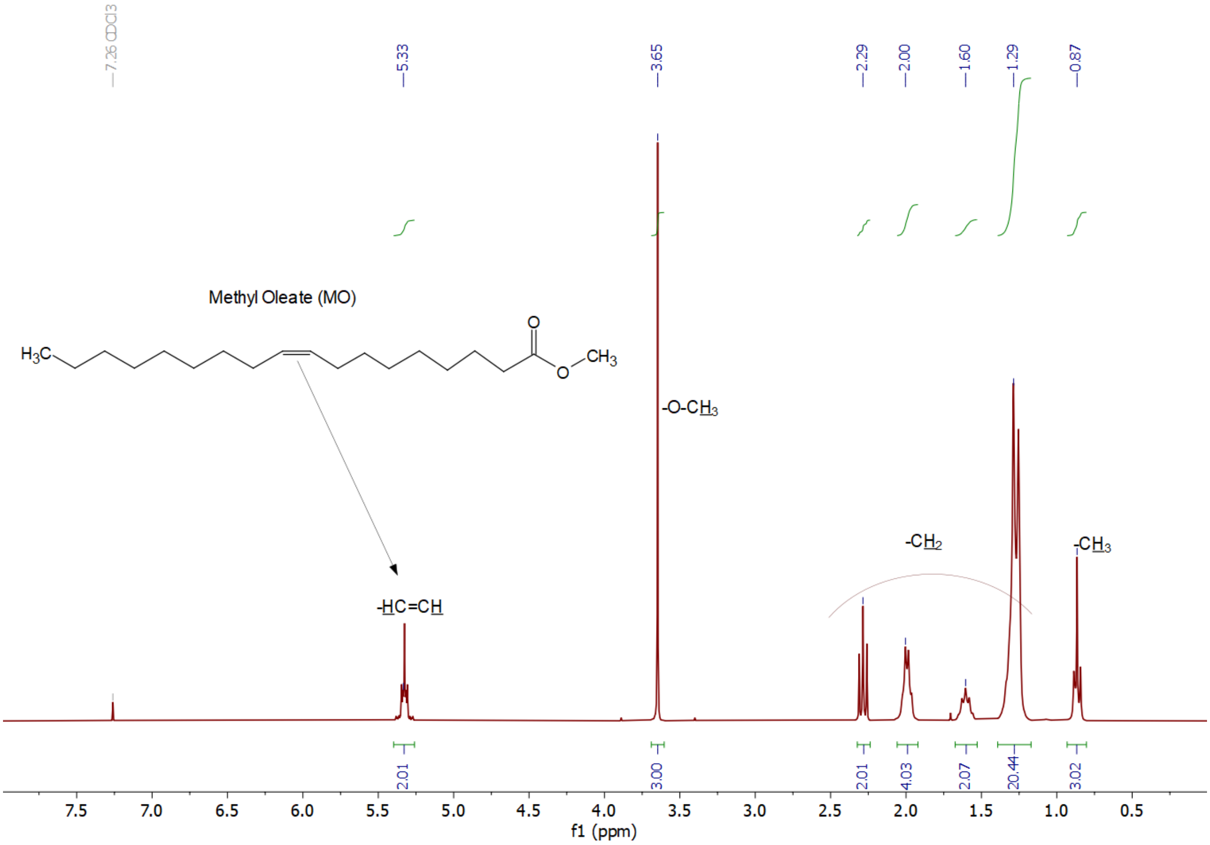

Figure S1. ^1H NMR spectrum of methyl oleate (substrate).

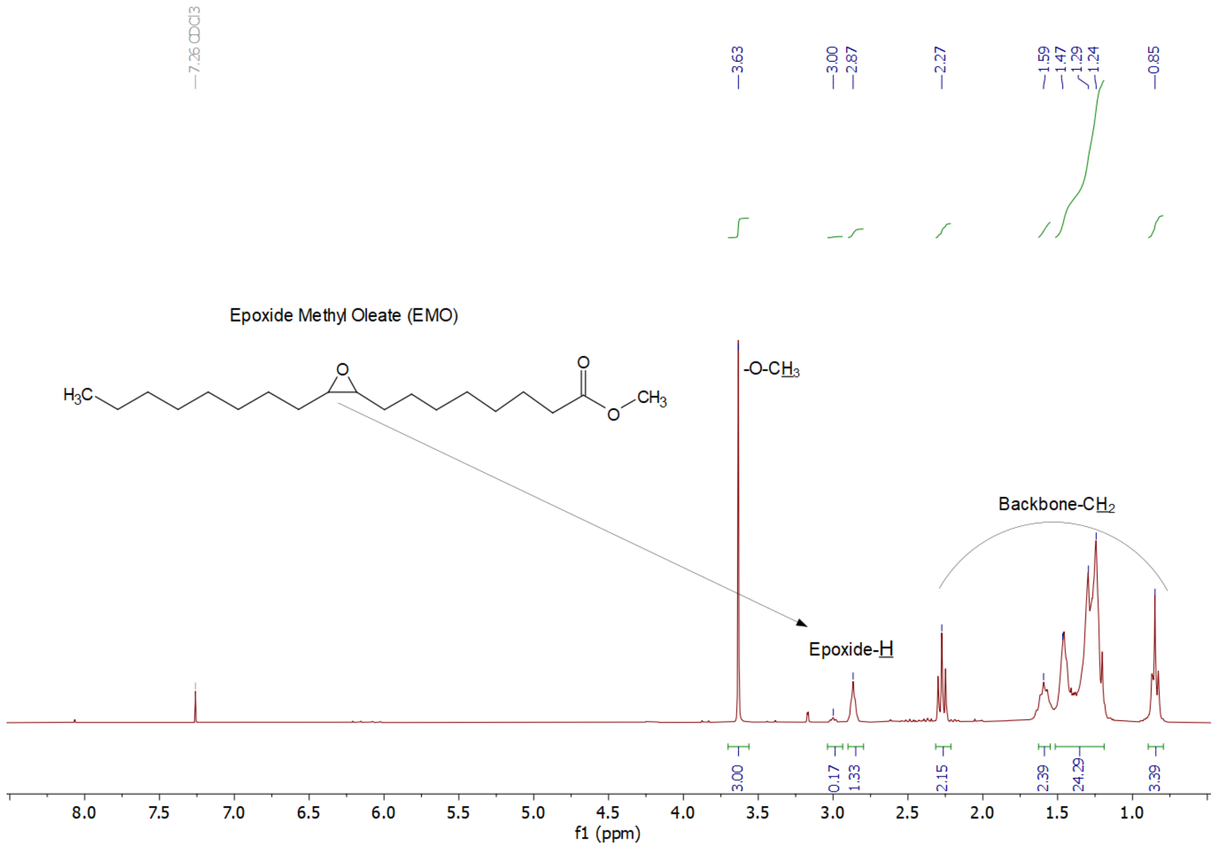

Figure S2. ^1H NMR spectrum of isolated epoxide methyl oleate (EMO) in $CDCl_3$. Reaction conditions: no added solvent, 60 °C, with ratio MO : TBHP : **5** = 1 : 2 : 0.02.

3. Quenching the Catalytic Reactions

Triphenylphosphine was used as a reducing agent for the oxidant TBHP to quench the epoxidation reaction chemically,[13] or physical quenching by diluting the withdrawn sample (50 µl) from the reaction mixture by adding 500 µl $CDCl_3$, then cooling the samples in ice (0 °C) until ^1H NMR measurement, both methods were tried to confirm the reliable results and the results are the same as shown in **Figure S3**.

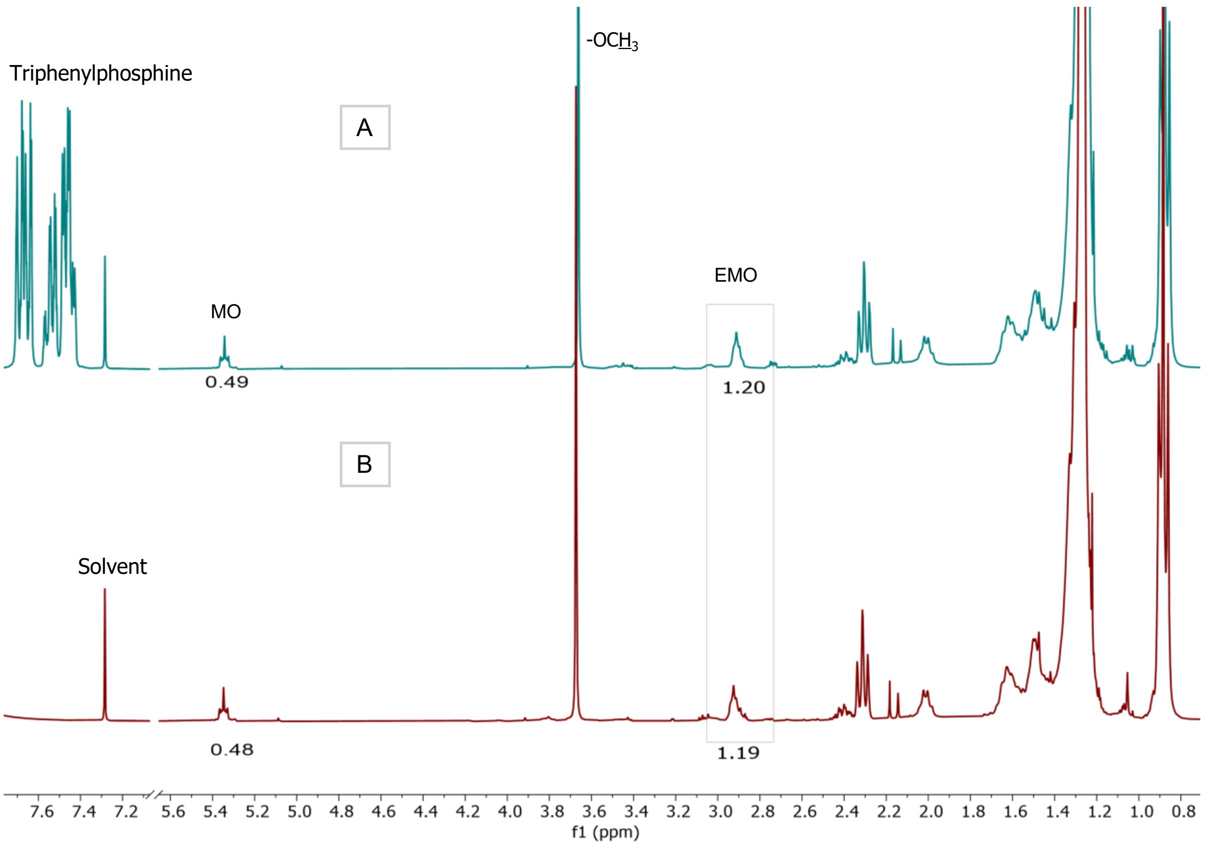

Figure S3. ^{1}H NMR spectra of the reaction mixture of methyl oleate epoxidation. Two different methods to quench the reaction before measuring. A) using triphenylphosphine to quench the reaction chemically. B) using dilution and ice to quench the reaction physically.

4. Effect of Time on the Epoxidation Performance

The effect of reaction time on the epoxidation of methyl oleate was evaluated by monitoring conversion, selectivity and yield. The best substrate conversion was achieved within 7 h, beyond which no significant improvement in product yield was observed. Notably, prolonged reaction times negatively impacted selectivity, likely due to secondary reactions such as epoxide ring opening or overoxidation. Therefore, 7 h was identified as the optimal reaction time, balancing complete conversion with maximum selectivity and minimizing undesired side processes.

5. Effect of Solvent on the Epoxidation

The effect of different solvents on the epoxidation of methyl oleate was evaluated under specific conditions (50 °C, 7 h) (**Table S4**). The system conducted without added solvent provided the highest selectivity (85%) and yield (71%) at 84% conversion, underscoring the advantage of neat conditions for this reaction. In contrast, reactions carried out in chloroform and acetonitrile showed lower selectivity (69% and 43%, respectively) and reduced yields (52% and 28%), indicating that these polar, coordinating solvents may interfere with catalyst activity or promote side reactions. Interestingly, toluene and decane maintained comparable conversion (84%, and 75%, respectively) but showed moderate selectivity (72%, and 76%) and yield (60%, and 57%), suggesting that nonpolar solvents exert less negative impact than polar, coordinating ones but still do not outperform the system conducted without added solvent. Overall, these findings highlight that the absence of solvent is optimal for maximizing both selectivity and yield in this epoxidation system, providing not only chemical efficiency but also aligning with green chemistry principles by eliminating unnecessary solvent use.

Table S4. Solvent screening

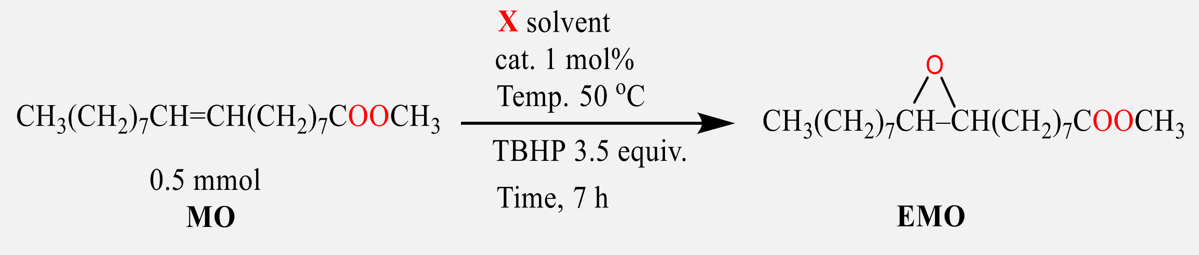

entry	Solvent	Conversion %	Selectivity %	Yield% (**EMO**)
**1**	**no added solvent***	**84**	**85**	**71**
2	Decane	75	76	57
3	Chloroform	76	69	52
4	Acetonitrile	66	43	28
5	Toluene	84	72	60

Catalyst **5** (1 mol%), methyl oleate (0.5 mmol, 170 µl). The Conversion %, selectivity % and EMO yield% was determined using ^{1}H NMR technique. Deuterated solvents (0.5 ml) were used except decane. *No added solvent but oxidant TBHP is in decane.

6. Effect of Temperature on Epoxidation

The influence of temperature on the conversion, selectivity, and yield of EMO is illustrated in **Figure S4** and summarized in **Table S5**. At elevated temperatures (70–80 °C), conversions were high (89–90%), but the modest selectivity (59–61%) limited the yield to 53–54%. Decreasing the temperature enhanced selectivity, with 50 °C providing the best overall compromise: 83% conversion and 67% selectivity, affording the maximum yield of 56%. Notably, process at 30 °C, although resulting in lower conversion (54%), gave an exceptionally high selectivity of 90%, with a respectable yield of 48%. This pronounced improvement in selectivity at reduced temperature suggests a kinetic preference for EMO formation under milder conditions, whereas elevated temperatures likely accelerate competing pathways. Taken together, these results indicate that 50 °C is optimal for maximizing isolated yield, while reactions at 30 °C may be advantageous when selectivity are the target.

Table S5. Temperature screening

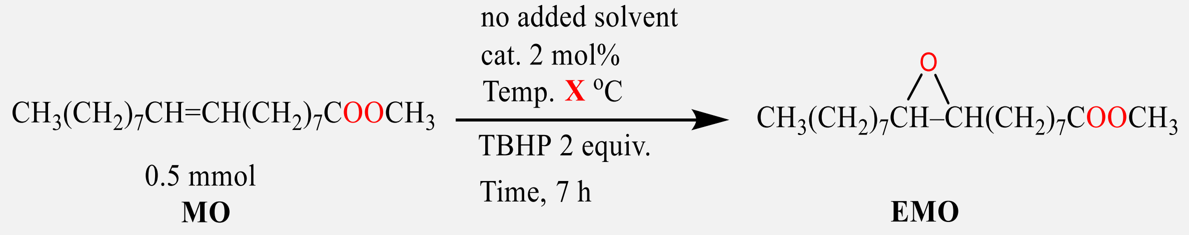

entry	Temperature °C	Conversion %	Selectivity %	Yield% (**EMO**)
1	80	89	61	54
2	70	90	59	53
3	60	83	66	55
**4**	**50**	**83**	**67**	**56**
5	40	71	71	50
**6**	**30**	**54**	**90**	**48**

Catalyst **5** (2 mol%), methyl oleate (0.5 mmol, 170 μl). Conversion, selectivity and EMO yield were determined using ^{1}H NMR spectroscopy.

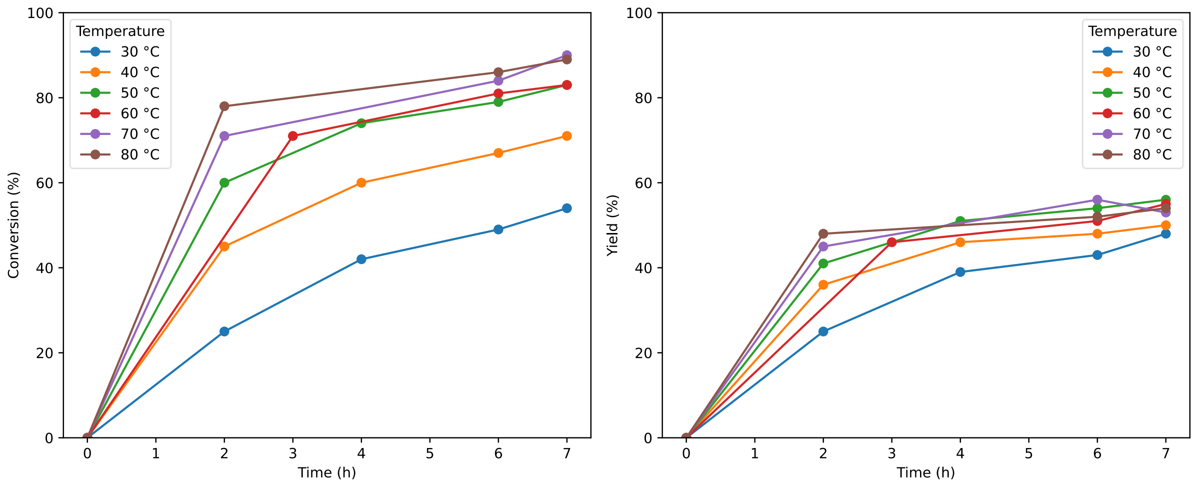

Figure S4. Effect of temperature on the conversion (left) and yield (right) in the epoxidation of methyl oleate (MO, 0.5 mmol). Catalyst **5** (2 mol%), TBHP (2 equiv.), Temperature (X °C), no added solvent. Conversion and epoxide (EMO) yield were determined by ^{1}H NMR spectroscopy.

7. Effect of Oxidant (TBHP) Loading on Epoxidation

The effect of varying *tert*-butyl hydroperoxide (TBHP) equivalents on the epoxidation catalysis of methyl oleate was systematically investigated (**Figure S5**; **Table S6**). At low oxidant levels (1.0 equiv), the conversion was limited to 66%, with modest selectivity (59%) and a yield of only 39%. The increases in TBHP steadily improved both conversion and selectivity; for example, 2.0 equiv. provided 83% conversion and 66% selectivity, affording a yield of 55%. At 3.0 equiv., the conversion rose to 90%, and the yield improved to 61%. The optimal performance was observed at 3.5 equiv. of TBHP, delivering nearly complete conversion (98%), enhanced selectivity (71%), and the highest yield of 70%.

Table S6. Varying the oxidant (TBHP) equivalents

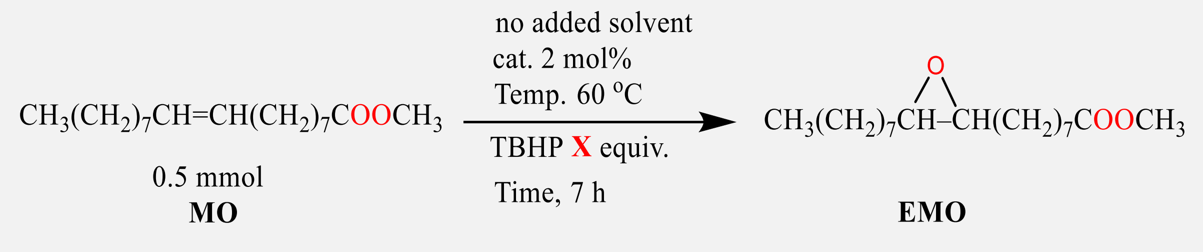

Entry	TBHP equiv.	Conversion %	Selectivity %	Yield % (EMO)
1	1.0	66	59	39
2	1.5	77	61	47
3	2.0	83	66	55
4	3.0	90	67	61
**5**	**3.5**	**98**	**71**	**70**

Catalyst **5** (2 mol%), methyl oleate (0.5 mmol, 170 µl). Conversion, selectivity and EMO yield were determined using ^{1}H NMR spectroscopy.

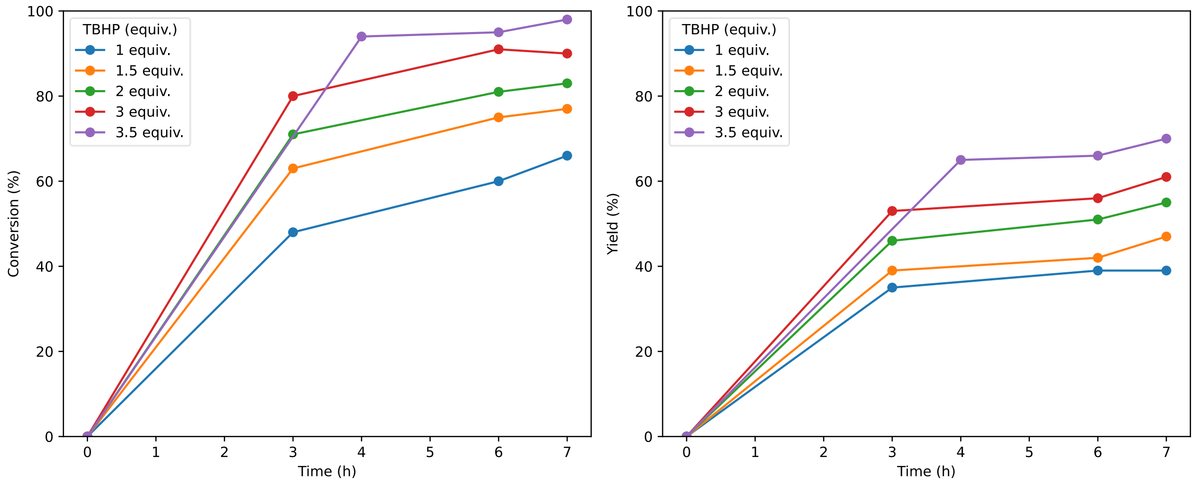

Figure S5. Effect of TBHP loading on the conversion (left) and yield (right) in the epoxidation of methyl oleate (MO, 0.5 mmol). Catalyst **5** (2 mol%), TBHP (X equiv.), no added solvent, 60 °C. Conversion and epoxide (EMO) yield were determined by ^{1}H NMR spectroscopy.

8. Effect of Catalyst Loading on Epoxidation

The influence of catalyst loading on the epoxidation of methyl oleate was systematically evaluated (**Figure S6**; **Table S7**). Increasing the catalyst load from 0.5 to 3 mol % led to a steady rise in conversion, from 79% at 0.5 mol% to 98% at 3 mol%. Selectivity, however, remained largely stable (~78–82%) up to 2 mol%, but dropped sharply to 68% at 3 mol%. Yield improved from 62% at 0.5 mol% to 69% at 1 mol%, then reached a maximum of 75% at 2 mol%; however, the marginal yield gain between 1 and 2 mol%. Considering the balance between catalytic efficiency, selectivity, and economic factors, 1 mol% is identified as the optimal catalyst loading, offering high conversion (88%) and competitive yield (69%) under milder conditions with improved process efficiency.

Table S7. Varying the catalyst loading

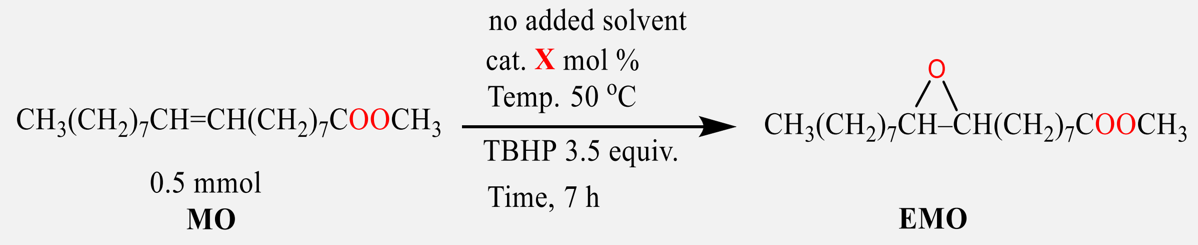

Entry	Catalyst mol%	Conversion %	Selectivity %	Yield% (**EMO**)
0	0	~3	0	0
1	0.5	79	78	62
**2**	**1**	**88**	**78**	**69**
3	2	92	82	75
4	3	98	68	67

Catalyst **5** (**X** mol%), methyl oleate (0.5 mmol, 170 µl). Conversion, selectivity and EMO yield were determined using ^{1}H NMR spectroscopy.

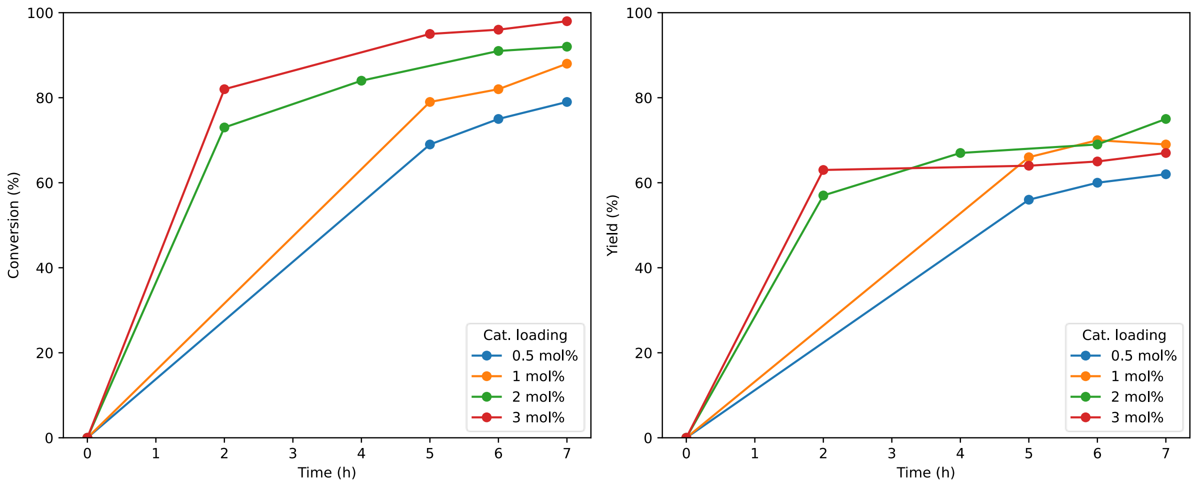

Figure S6. Effect of catalyst loading on the conversion (left) and yield (right) in the epoxidation of methyl oleate (MO). Catalyst **5** (X mol%), methyl oleate (0.5 mmol), TBHP (3.5 equiv.), no added solvent, 50 °C. Conversion and epoxide (EMO) yield were determined by ^{1}H NMR spectroscopy.

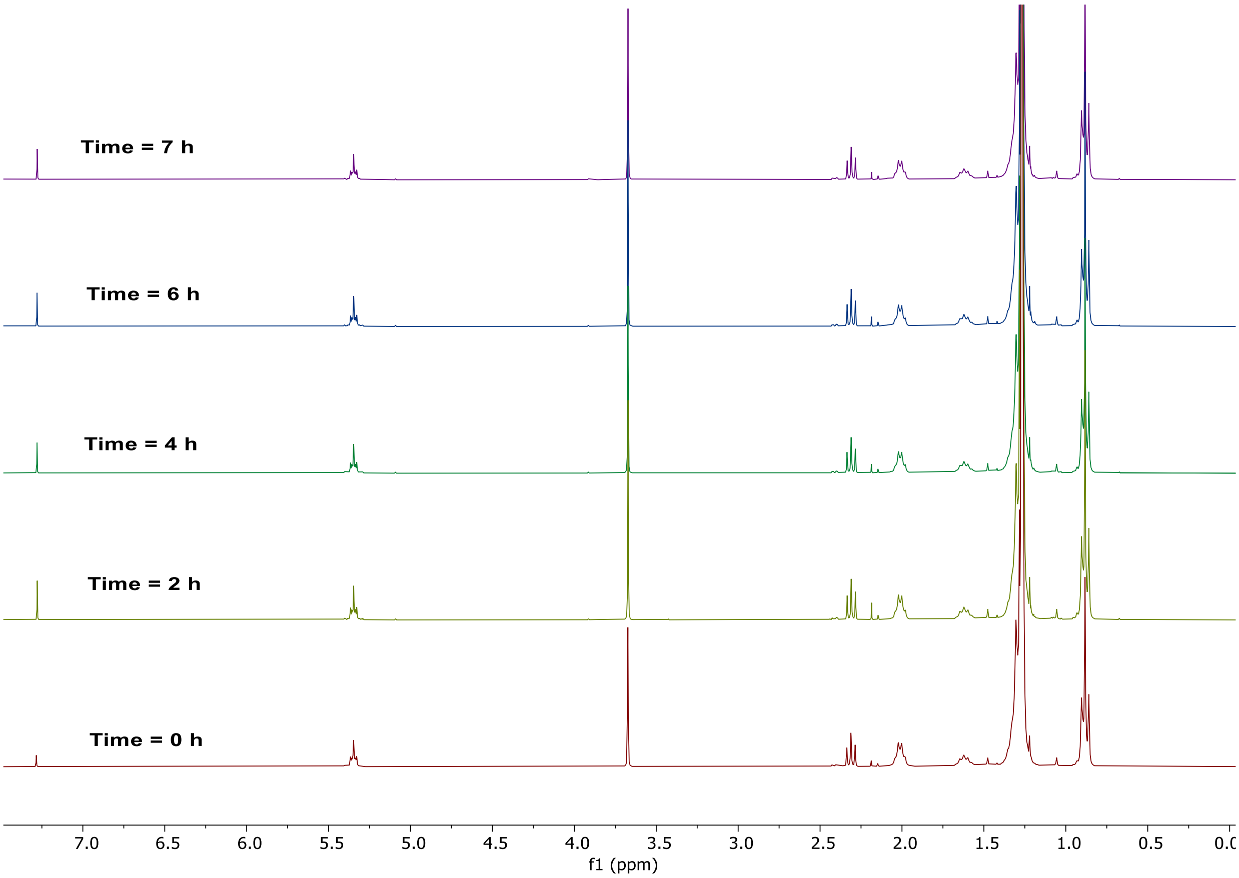

Figure S7. ^{1}H NMR spectra in deuterated chloroform for the reaction mixture (epoxidation of methyl oleate) under the following conditions: without catalyst, no added solvent, 50 °C, 3.5 equiv. oxidant (TBHP) vs. substrate (MO).

6. Catalyst Screening

Table S8. Screening the performance of catalysts for epoxidation of MO at RT and 50 °C.

**Catalyst**	**1**		**4**		**$VOSO_4$**	**2**		**5**		**3**	
	RT	50 °C	RT	50 °C	50 °C	RT	50 °C	RT	50 °C	RT	50 °C
**Conv. %**	81	83	73	85	93	89	81	87	84	93	94
**Select. %**	59	60	65	65	63	72	79	76	85	98	85
**Yield %**	48	50	47	55	59	64	64	66	71	91	80

Conversion, selectivity and yield were determined by ^{1}H NMR spectroscopy.

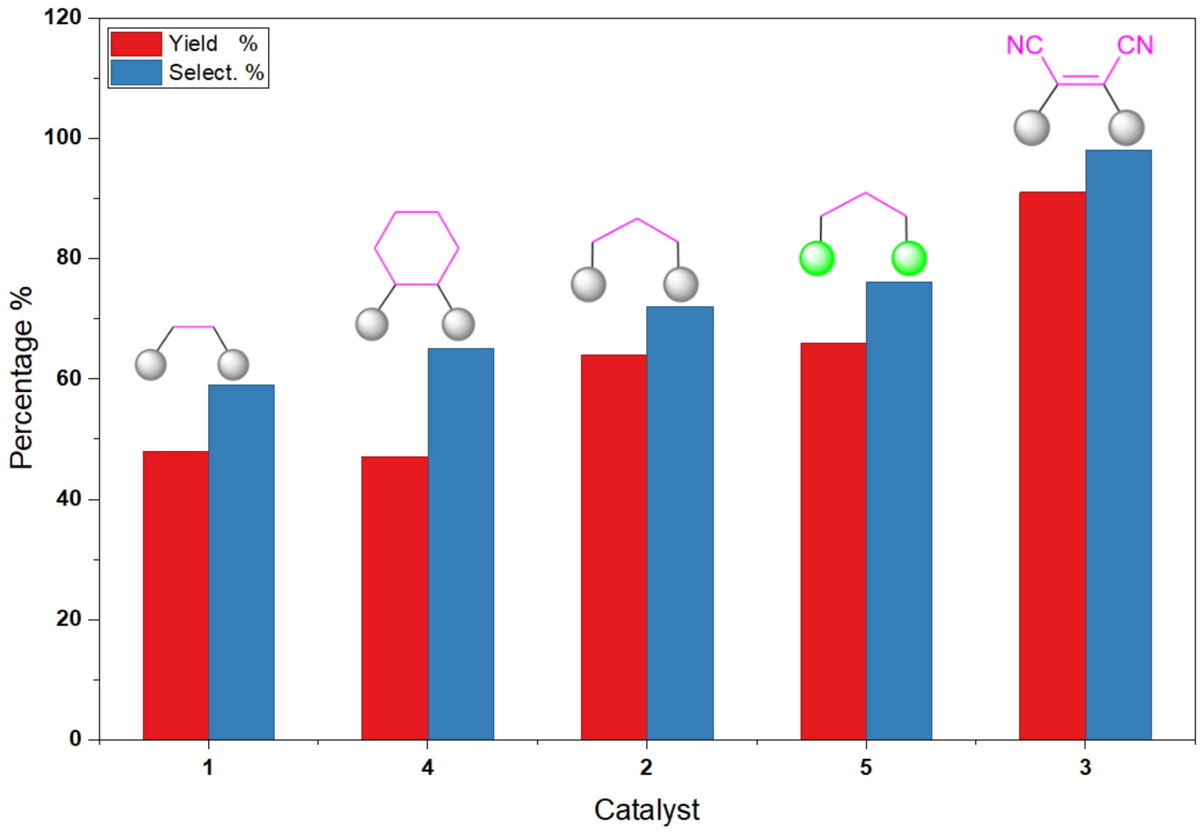

Figure S8. Structure of catalysts **1-5** vs selectivity and yield for epoxidation at RT.

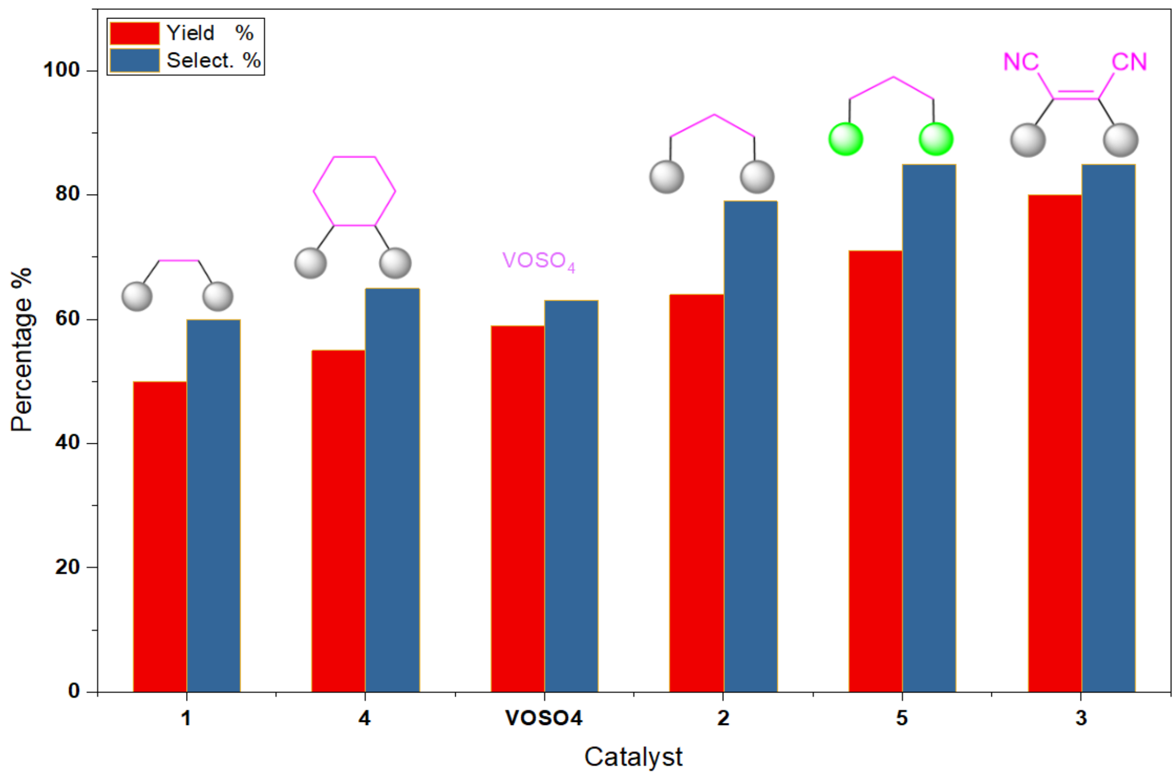

Figure S9. Structure of catalysts **1-5** vs selectivity and yield for epoxidation at 50 °C.

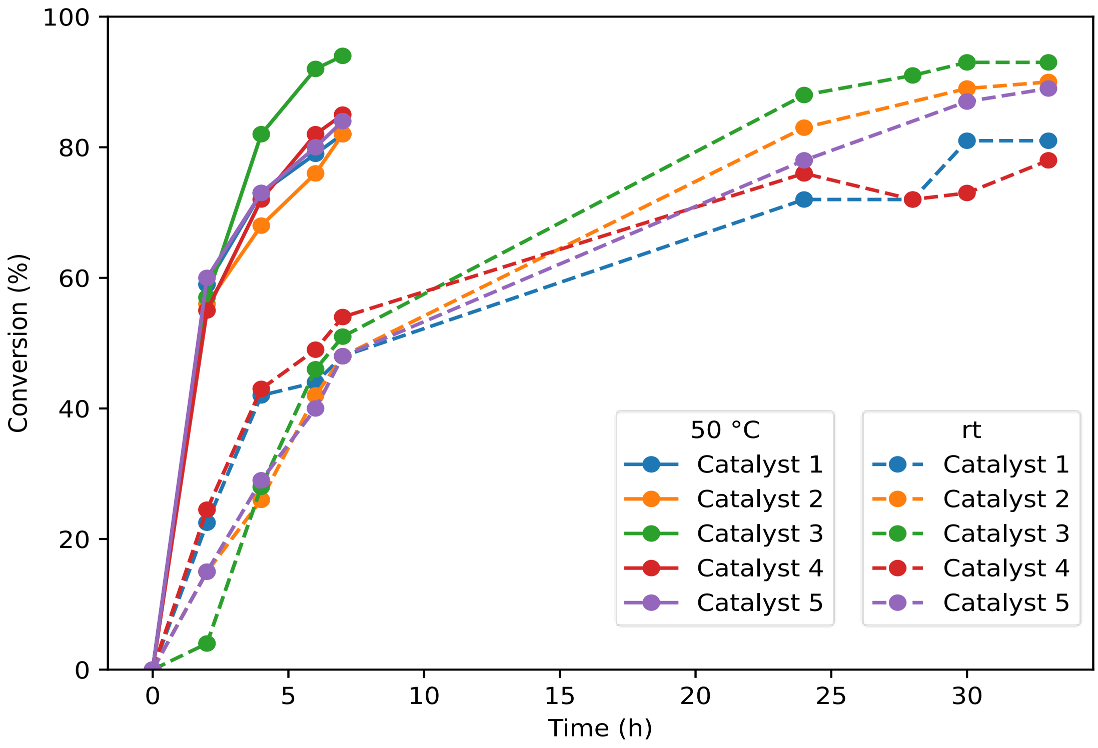

Figure S10. Time-dependent profiles of Conversion for the epoxidation of methyl oleate (MO, 0.5 mmol) catalyzed by catalyst **1–5** (1 mol%) using (TBHP, 3.5 equiv.) at 50 °C (solid lines) and room temperature (rt, dashed lines). Conversion was determined by ^{1}H NMR.

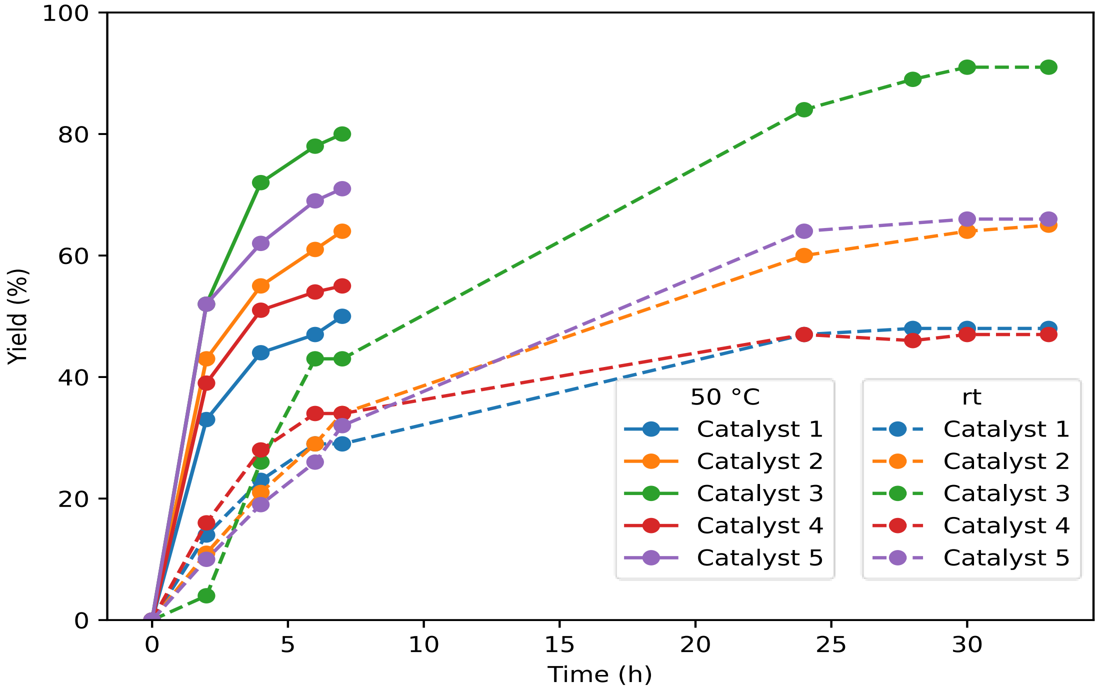

Figure S11. Time-dependent profiles of yield (EMO) for the epoxidation of methyl oleate (MO, 0.5 mmol) catalyzed by catalyst **1–5** (1 mol%) using (TBHP, 3.5 equiv.) at 50 °C (solid lines) and room temperature (rt, dashed lines). Yield was determined by ^{1}H NMR.

7. Mechanistic Study

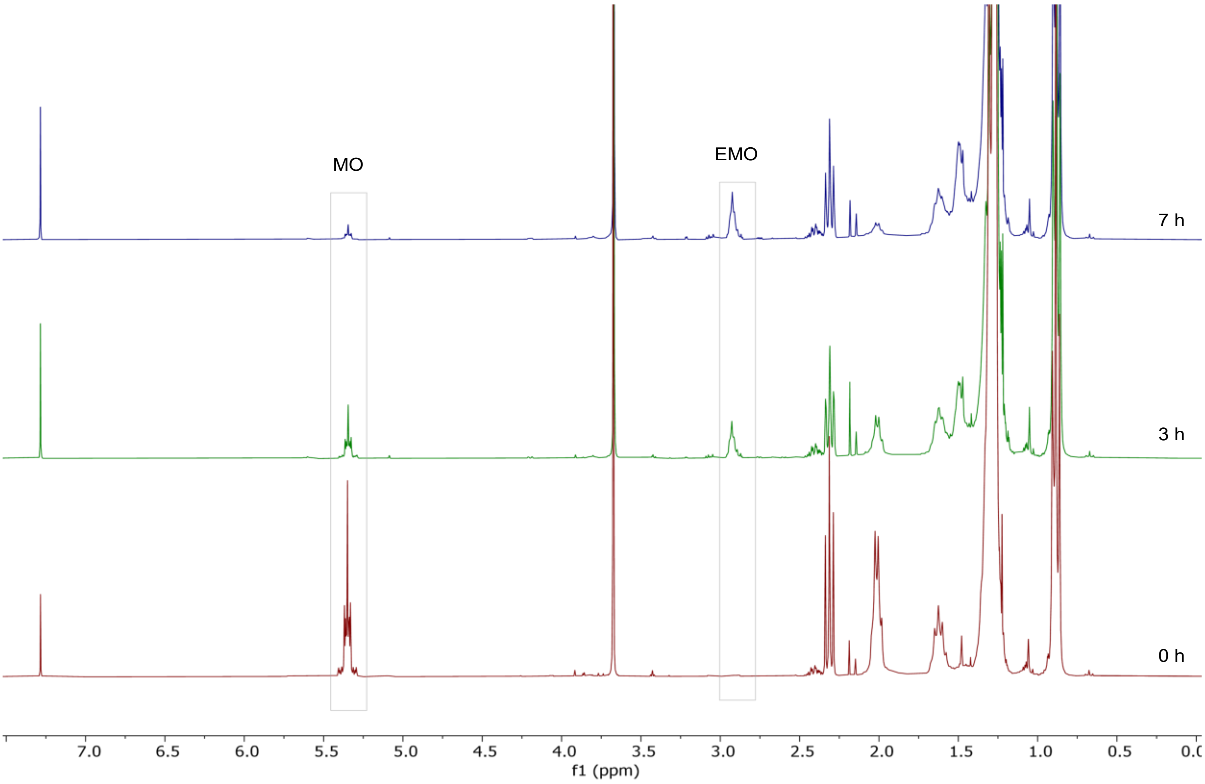

Figure S12. ^{1}H NMR spectra of the epoxidation of methyl oleate (MO) using catalyst **5** without BHT over a 7-hour period.

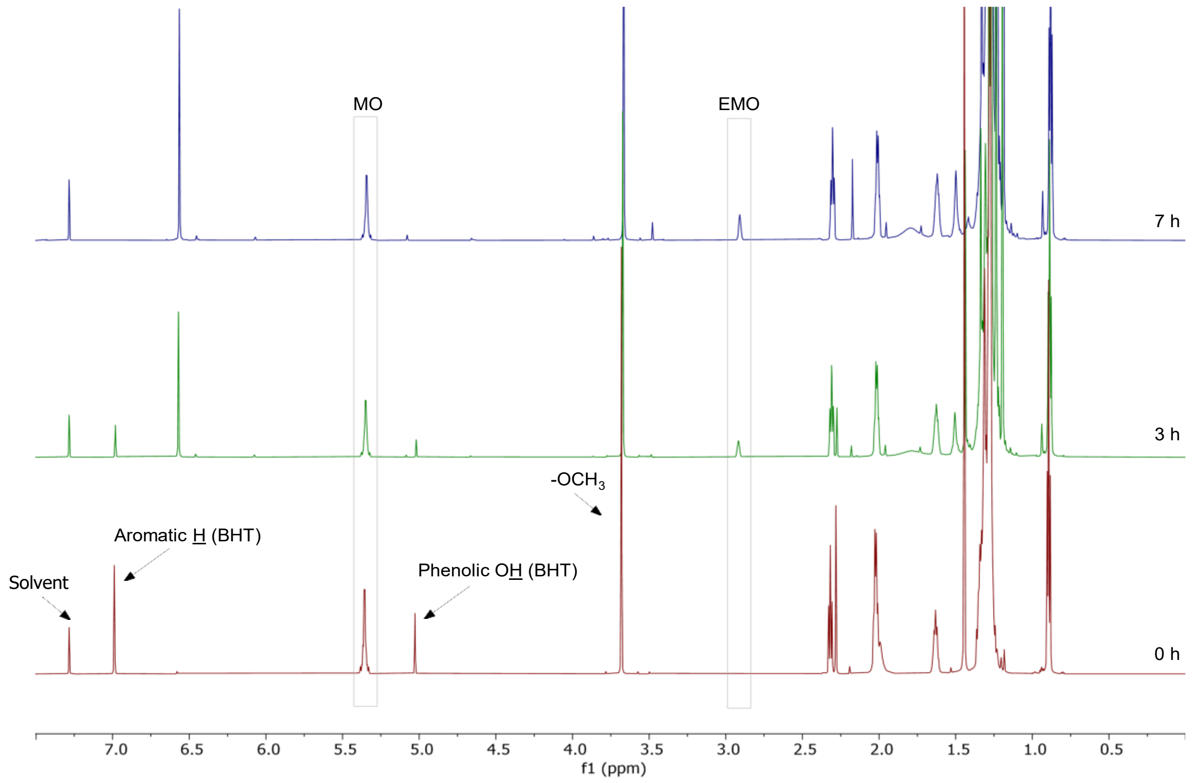

Figure S13. ^{1}H NMR spectra of the epoxidation of methyl oleate (MO) using catalyst **5** in the presence of BHT (1 equiv.) as a radical scavenger over a 7-hour period.

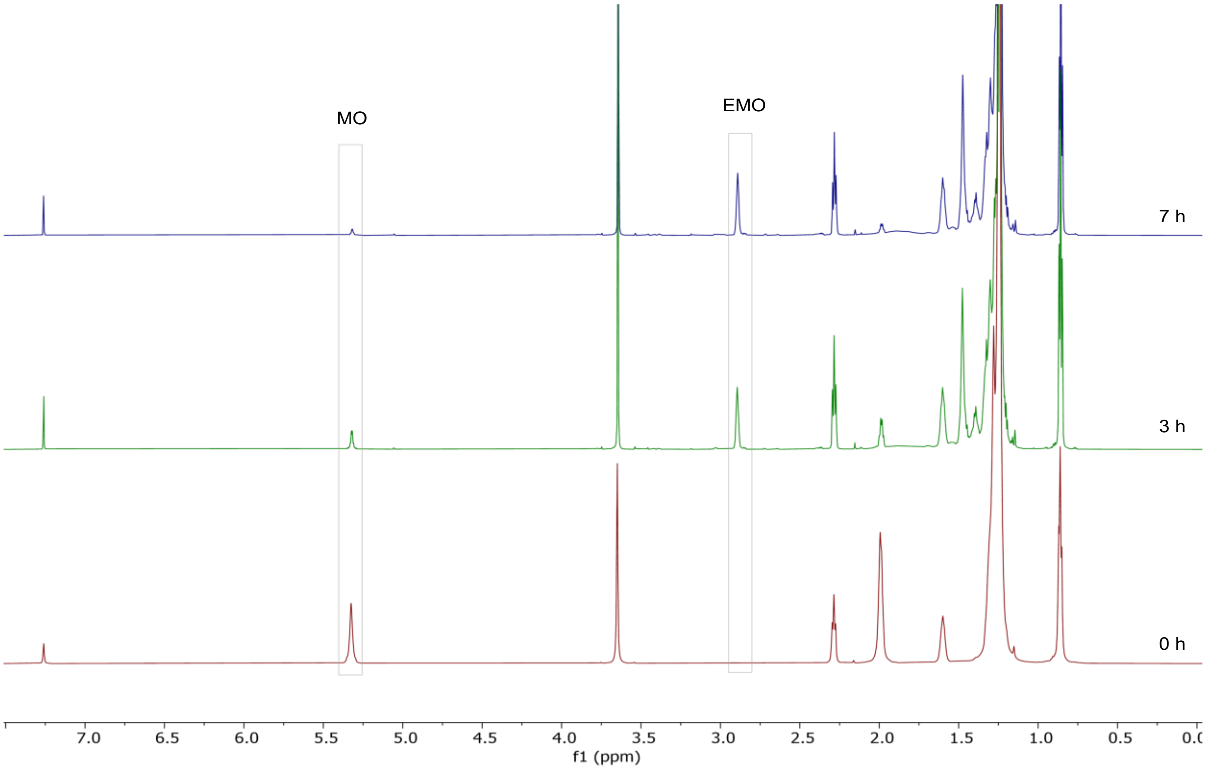

Figure S14. ^{1}H NMR spectra of the epoxidation of methyl oleate (MO) using catalyst **3** without BHT over a 7-hour period.

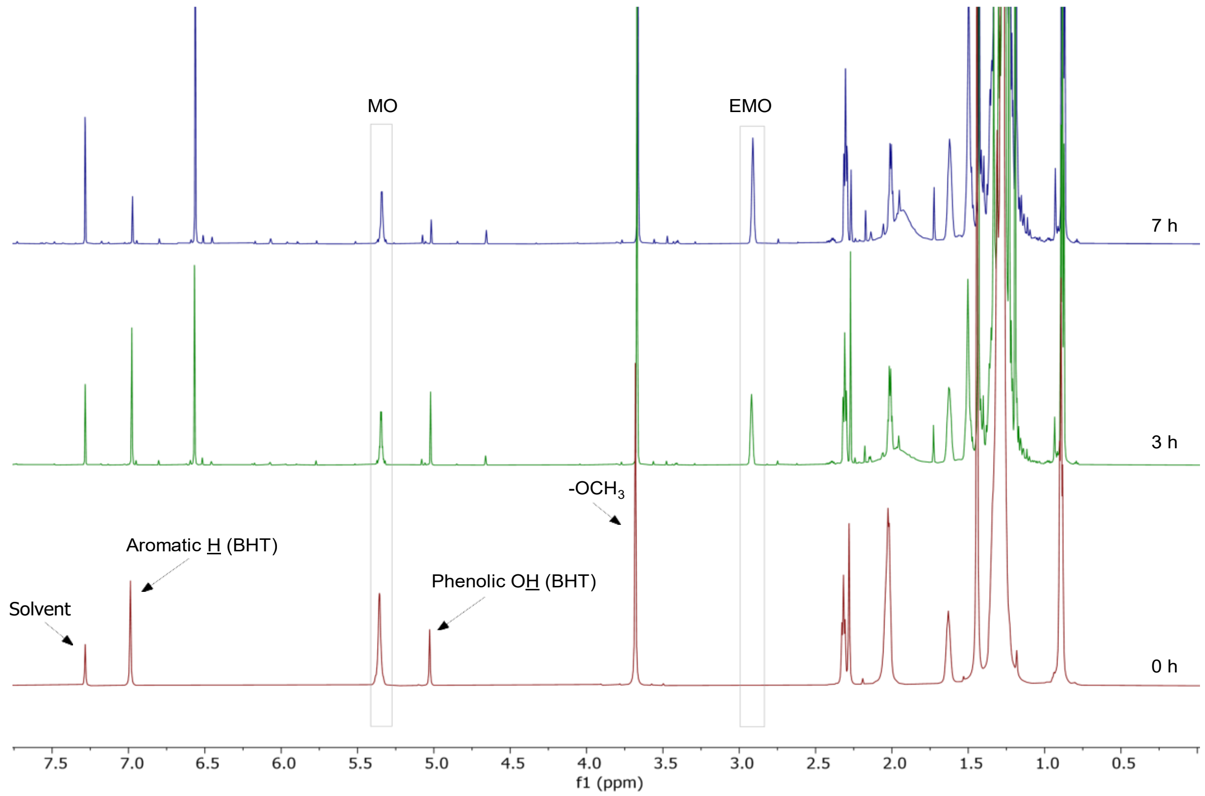

Figure S15. ^{1}H NMR spectra of the epoxidation of methyl oleate (MO) using catalyst **3** in the presence of BHT (1 equiv.) as a radical scavenger over a 7-hour period.

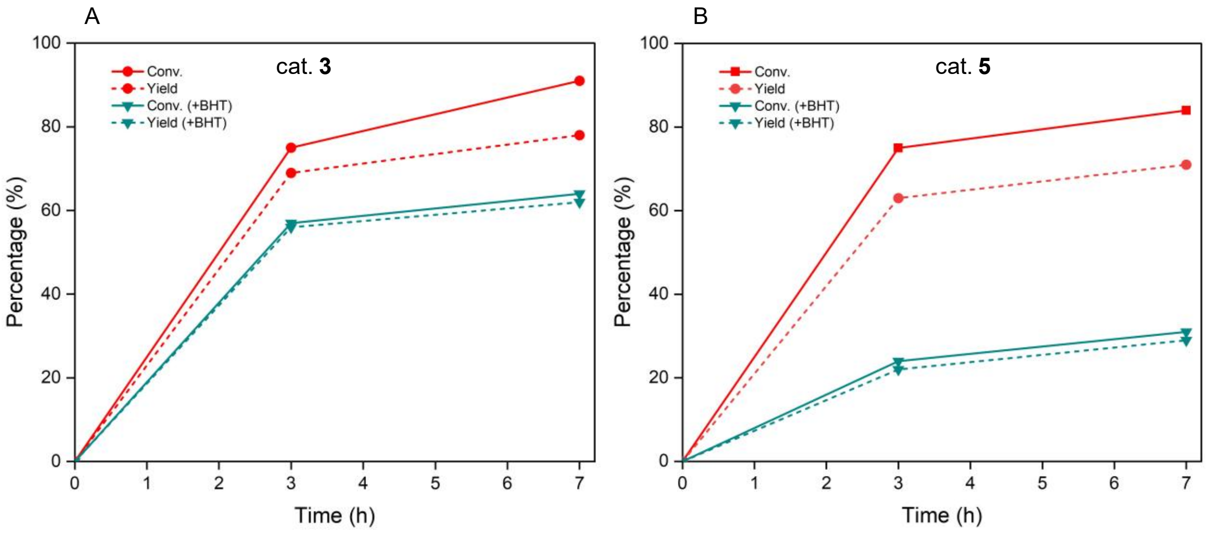

Figure S16. Effect of BHT (1 equiv.) on the epoxidation of methyl oleate under the optimized reaction conditions. (A) cat. **3**. (B) cat. **5**. Conversion and epoxide yield are shown as a function of reaction time in the absence and presence of BHT.

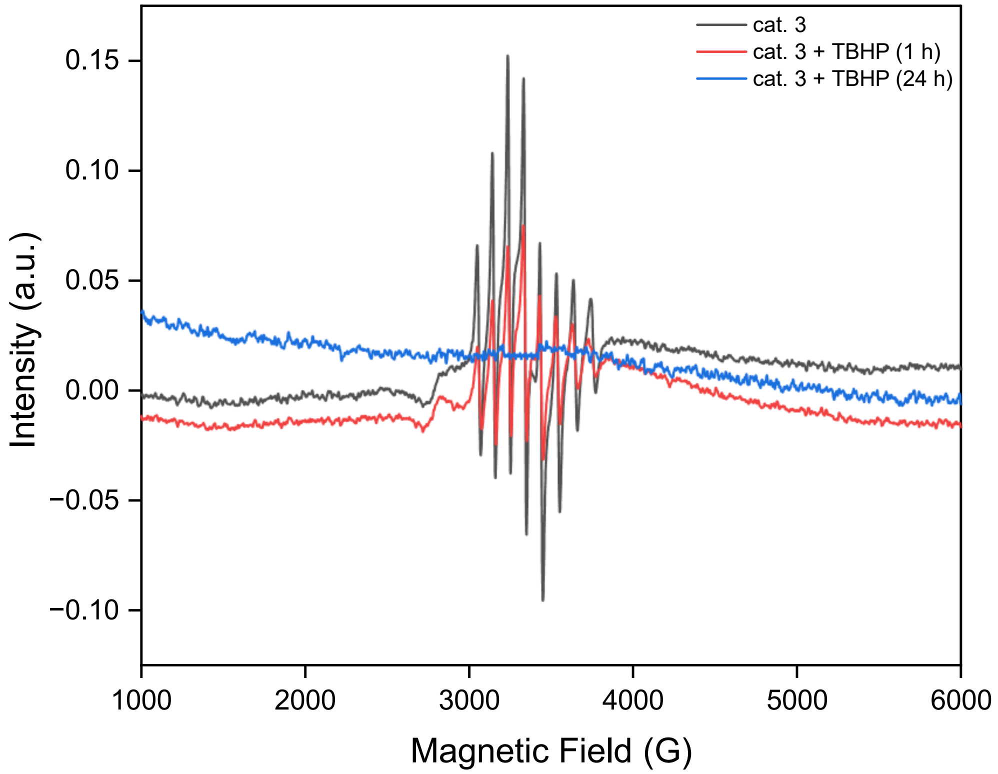

Figure S17. Background-subtracted X-band EPR spectra of cat. **3** recorded in $CHCl_3$ at room temperature before and after addition of TBHP.

8. Kinetic Study

Kinetics Studies Using UV-Vis Spectroscopy in $CDCl_3$

Sample preparation: All solutions were prepared and used freshly. Stock solutions of catalysts **1-5** were prepared in $CHCl_3$ (0.15 mM) but the stock solution of TBHP in decane was used as purchased (5.64 M – the concentration was determined by iodometric titration). These stock solutions of catalysts **1-5** and TBHP were used for all measurements. For the determination of the pseudo-first-order rate constants, all data sets for 0.05 mM catalyst **2** were measured using 150, 175, and 200 equiv. of TBHP, whereas catalyst **5** was measured using 200, 225, and 250 equiv. of TBHP.

Kinetic analysis of V(IV) oxidation: All kinetic measurements were carried out using a large excess of TBHP (pseudo-first-order conditions). Typically, a stock solution of TBHP (5.64 M in decane) added directly to a thermostatic UV quartz cell (30 °C) containing an appropriate

amount of a solution of the oxovanadium catalysts in $CHCl_3$ (0.15 mM) to obtain a total volume of 3 mL with a final concentration of catalysts equal to (0.05 mM). The product formation was monitored against time by following absorbance changes at a specific wavelength in the range 300–500 nm, using a spectrophotometer. The delaying time before measuring is around 0.5 min. The reactions were monitored by Agilent Cary60 Uv-Vis Spectrophotometer equipped with a VWR thermostat using WinUv software and thereof "Scan" program. The reactions were scanned in the range λ = 800 – 200 nm and the oxidation of catalysts by TBHP was studied by observing the change in absorbance of the reaction product.

The pseudo-first-order rate constants (k_{obs}) were determined by applying the Guggenheim linearization **eq (S1)**, while the oxidation reaction rates (k) were obtained from the slope of the plots of k_{obs} vs [TBHP]. First, the logarithmic values of the absorbance changes at the given wavelength (λ) were plotted against time to extract the slopes. The resulting slopes were then plotted versus the oxidant concentration to determine the pseudo-first-order rate constants. For each TBHP concentration, the experiments for the determination of k_{obs} were repeated three times.

$$\ln[A(t+\Delta t) - At] = \ln[A_0 - A_\infty] \cdot (e^{-kt} - 1) - kt \qquad \textbf{eq (S1)}$$

The Gibbs free energy ($\Delta G^\ddagger$) for catalysts **2** and **5** with (TBHP) was calculated by applying Eyring's **eq (S2)**.

$$\Delta G^\ddagger = - RT\ln(k_{obs}h/k_BT) \qquad \textbf{eq (S2)}$$

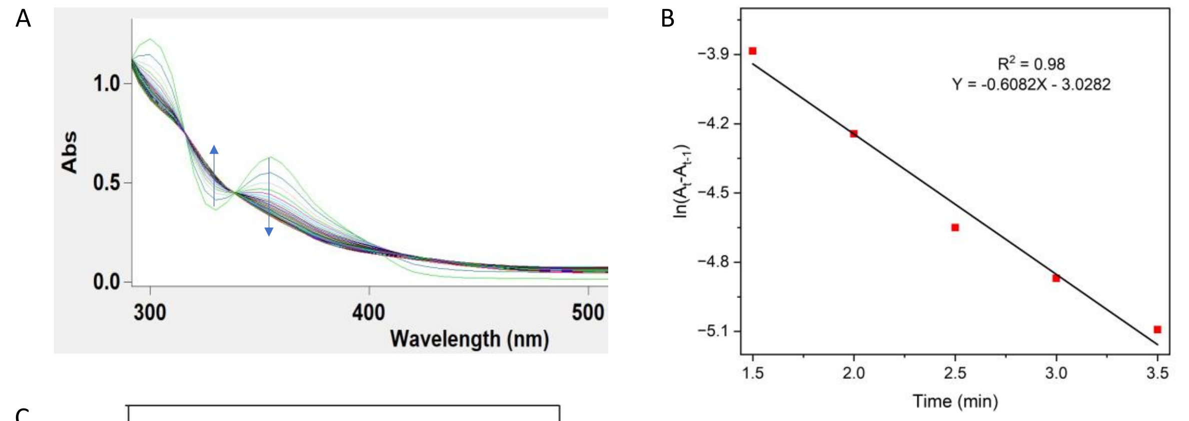

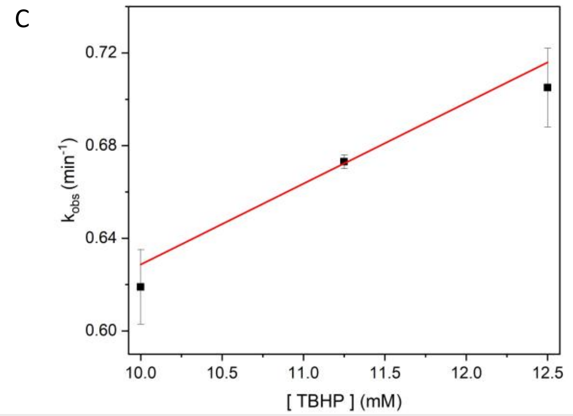

D

	k (min^{-1})	ΔG‡ (kJ mol^{-1})
cat. **2** (*P*-H)	0.1816	88.91
cat. **5** (*p*-OMe)	0.0344	93.10

Figure S18. A) UV–Vis spectra of the reaction of 0.05 mM catalyst **5** with 200 equiv. of oxidant TBHP in $CDCl_3$. **B**) Guggenheim linearization for catalyst **5** in $CDCl_3$, conc.= 0.05 mM + **200** equiv. TBHP. **C**) Determination of the oxidation reaction rate k for complex **5** in $CDCl_3$. Each measurement was repeated three times. **D**) The Gibbs free energy of catalysts **2** and **5** for oxidation reaction.

9. Computational Study

Density functional theory (DFT) calculations were performed with Gaussian 09.[14] Geometries were optimized using Becke, 3-parameter, Lee-Yang-Parr hybrid functional (B3LYP) under two protocols: (i) second-generation triple-zeta valence basis set (def2TZVP) on all atoms and (ii) mixed basis set, employing def2TZVP for light atoms and the Stuttgart–Dresden (SDD) basis set for vanadium.[15,16] Empirical dispersion effects were accounted for using Grimme's D3 correction, ensuring a proper description of weak interactions.[17] Harmonic frequency analysis at the same level verified minima (no imaginary frequencies). Natural population analyses (NBO) were carried out on optimized structures; metal and oxo charges are reported as q(V) and q(O), and the polarization descriptor is defined as $\Delta q = q(V) - |q(O)|$.[18]

Experimental conversion, selectivity, and yield (RT and 50 °C) were correlated with Δq by ordinary least-squares fits (reporting R^2).

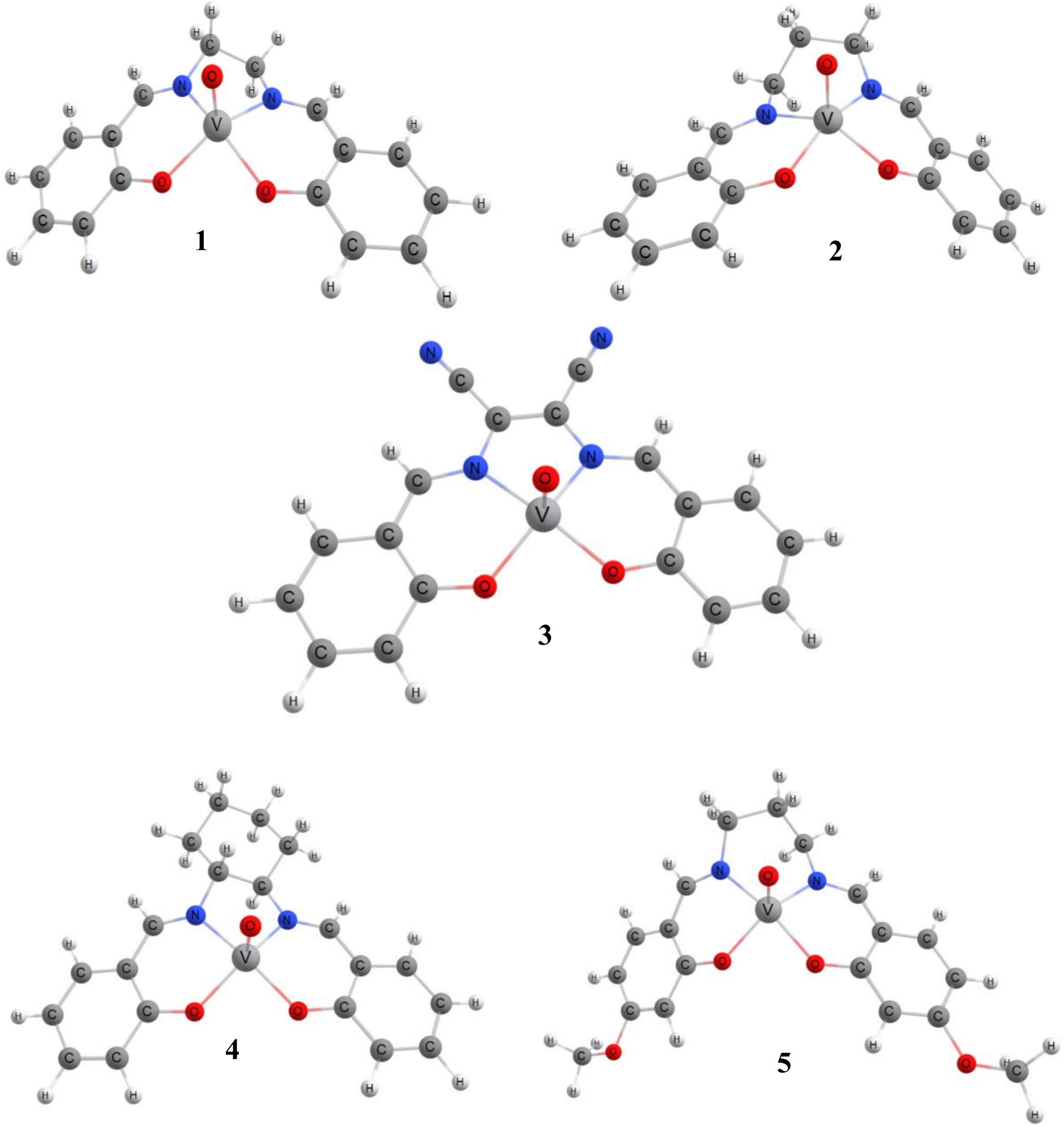

Figure S19. Optimized structures of catalysts **1-5** using B3LYP/(def2TZVP, SDD).

Table S9. Selective NBO charge distribution of catalysts **1-5**.

Catalyst	B3LYP/def2TZVP. NBO			B3LYP/(def2TZVP, SDD). NBO		
	q(V)	q(O)	Δq	q(V)	q(O)	Δq
**1**	0.801	-0.383	0.418	0.878	-0.401	0.477
**2**	0.830	-0.387	0.443	0.908	-0.406	0.502
**3**	0.819	-0.356	0.463	0.891	-0.371	0.520
**4**	0.810	-0.389	0.421	0.887	-0.407	0.480
**5**	0.831	-0.391	0.440	0.908	-0.410	0.498

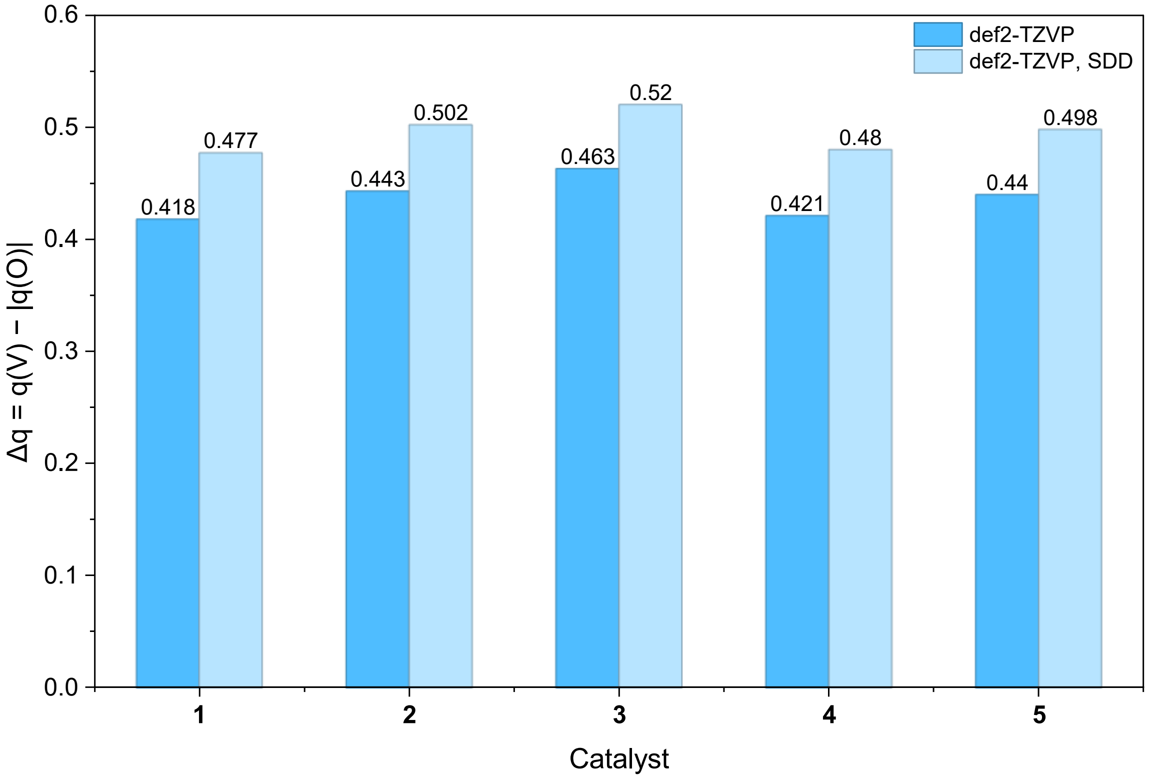

Figure S20. V═O polarization Δq of catalysts in activity order (3 > 5 > 2 > 4 > 1) using B3LYP for comparing (def2TZVP) and mixed basis set (def2TZVP, SDD).

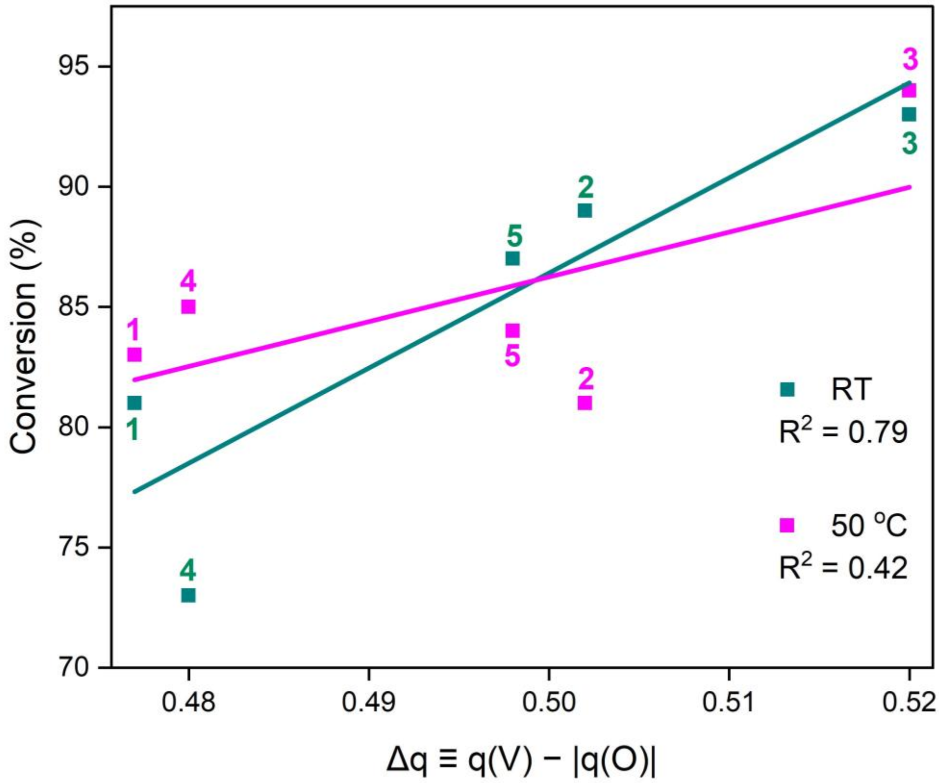

Figure S21. Correlation of substrate conversion with V═O polarization (Δq) obtained from NBO charges at two different temperature RT and 50 °C using B3LYP/(def2TZVP, SDD).

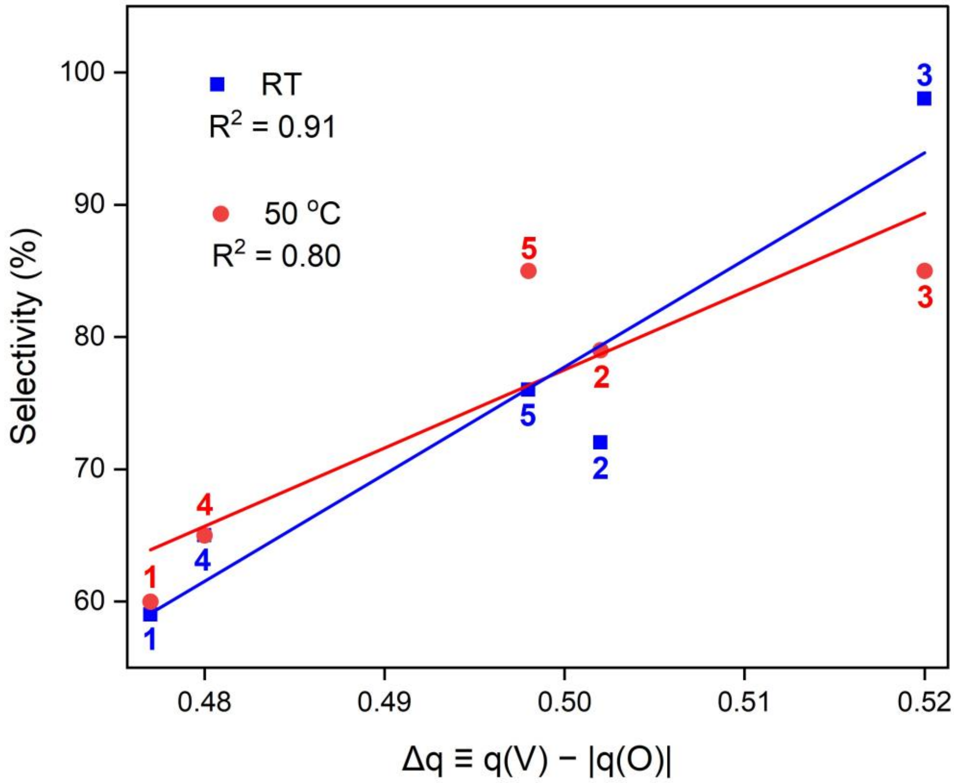

Figure S22. Correlation of selectivity with V═O polarization (Δq) obtained from NBO charges at two different temperature RT and 50 °C using B3LYP/(def2TZVP, SDD).

	Alpha-SOMO	Beta-LUMO	SOMO - LUMO (eV)
cat. **3**	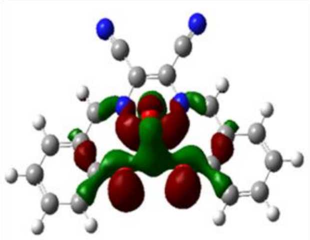	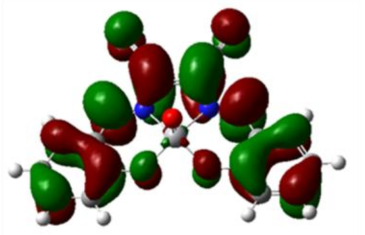	-3.697<br>ΔE = -2.825<br>-6.522
cat. **5**	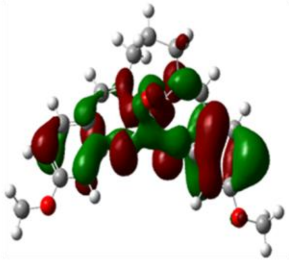	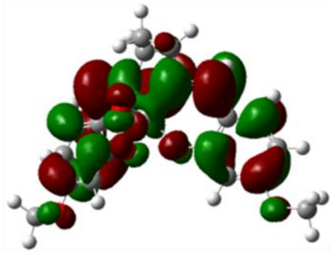	-1.821<br>ΔE = -3.786<br>-5.607
cat. **2**	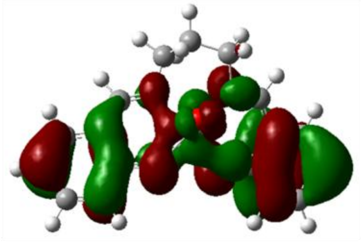	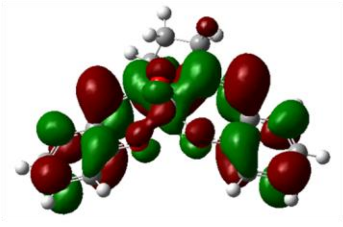	-2.116<br>ΔE = -3.644<br>-5.760
cat. **4**	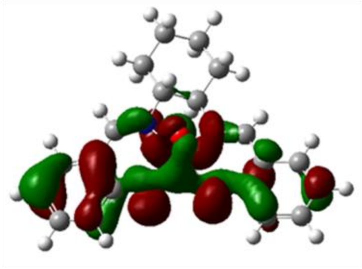	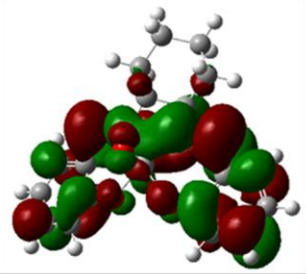	-2.082<br>ΔE = -3.586<br>-5.668
cat. **1**	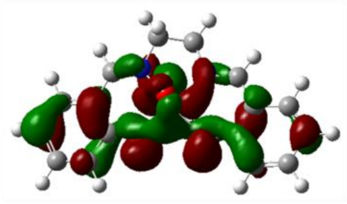	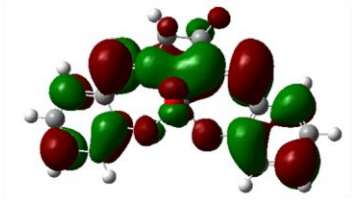	-2.231<br>ΔE = -3.528<br>-5.759

Figure S23. Frontier molecular orbitals (α-SOMO and β-LUMO) of catalysts **1-5** using B3LYP/(def2TZVP, SDD).

10. Crystal Structure

The single crystal X-ray diffraction measurement was carried out on a Bruker D8 Venture single crystal X-ray diffractometer equipped with *IuS* II microfocus Cu Kα (λ = 1.54178 Å) source and PHOTON II detector. The crystal was kept at 120 K during data collection. The structure was solved with the SHELXT structure solution program,[19] using Intrinsic Phasing and refined with the XL,[20] refinement package using Least Squares minimization implemented in Olex2.[21] The crystallographic data of the structure can be found in CCDC with the depository numbers 2531520.

Suitable single crystals of [VO(L_5)(DMSO)] (**5**) were grown from a saturated DMSO solution. The crystal structure of **5** was refined in the monoclinic space group *Pn* (SG: 7) symmetry with two independent oxovanadium molecules in the asymmetric unit hosting the lattice water molecules in the channel (**Figure S24**). The DMSO molecule coordinated to the V2 atom is split into two positions with partial occupancy of 0.73/0.27. Since the *Pn* is a non-centrosymmetric space group the Flack parameter was also refined, resulting in 0.26. The V centers have distorted octahedral coordination geometries defined by the N and O-donor atoms of the chelating ligand together with terminal oxygen and DMSO molecule. The distortion from ideal octahedral coordination is primarily related to the ligand bite constraints and asymmetric donor environments. Similar octahedral distortion geometry is also observed in complex **1**,[9] and N,O Schiff-base modified vanadium complexes.[11] The 3D supramolecular feature of catalyst **5** is realized through an extensive H-bonding, which involves co-crystallized water molecules and **L5** ligand oxygen atoms (**Figure S25**). The oxovanadium molecules assemble to form 1D channels propagating along [010] direction, accommodating lattice water molecules. These channels stack along [100] in …*AB*… fashion resulting in 3D supramolecular structure. All the crystallographic information on data collection, refinement, atomic coordinates, bond distances and angles were presented in (**Table S10-S15**).

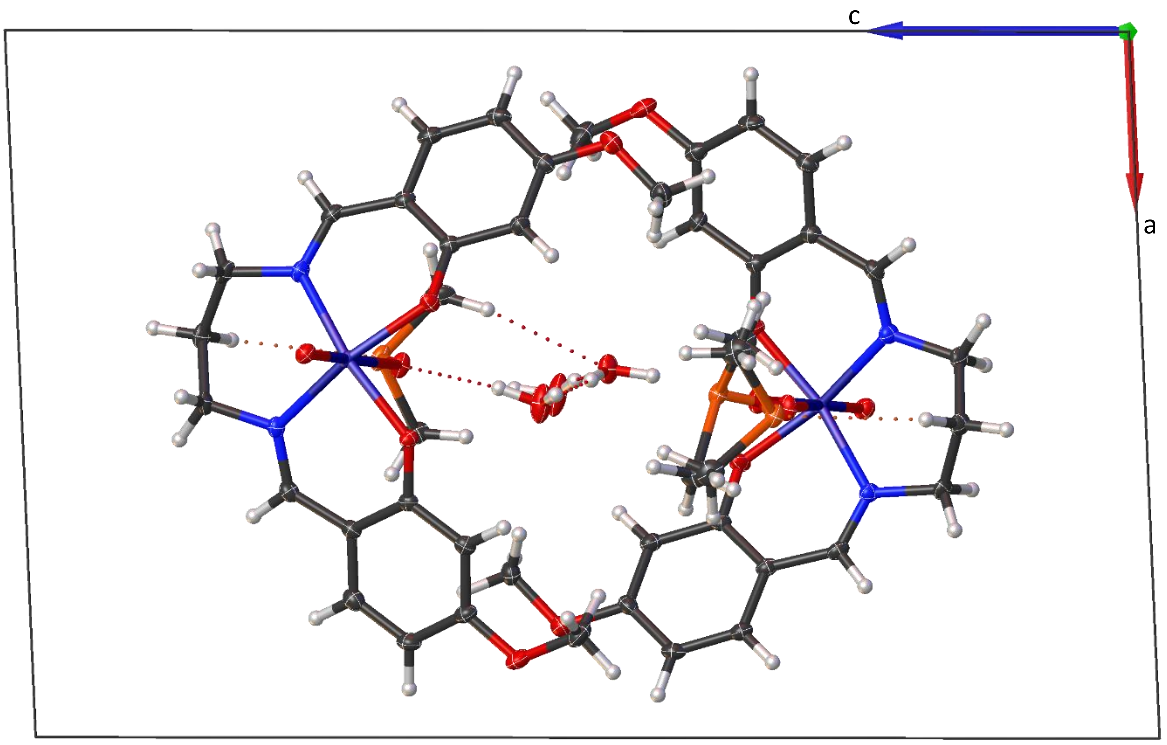

Figure S24. Asymmetric unit of the crystal structure of catalyst **5**.

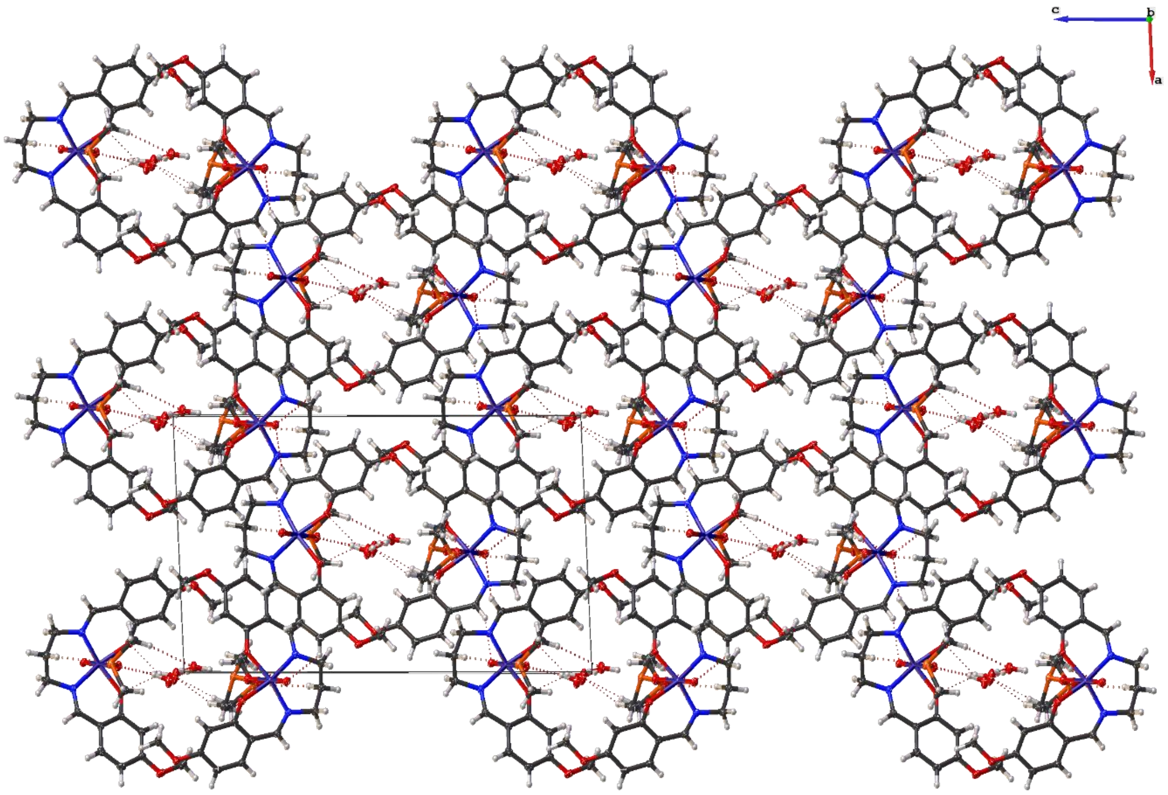

Figure S25. 1D channels running parallel to [010] direction accommodating water molecules. These channels stack in …AB… style along [100] to form 3D supramolecular structure.

Table S10. Crystal data and structure refinement for [VO(L_5)(DMSO)] (**5**).

Empirical formula	$C_{42}H_{58}N_4O_{15}S_2V_2$
Formula weight	1024.92
Temperature/K	120.00
Crystal system	monoclinic
Space group	*Pn*
a/Å	13.6747(4)
b/Å	7.7212(2)
c/Å	21.7194(7)
α/°	90
β/°	92.5730(10)
γ/°	90
Volume/Å^3	2290.93(12)
Z	2
ρ_{calc}g/cm^3	1.486
μ/mm^{-1}	4.889
F(000)	1072.0
Crystal size/mm^3	0.1 × 0.1 × 0.1
Radiation	Cu Kα (λ = 1.54178 Å)
2Θ range for data collection/°	7.49 to 149.152
Index ranges	$-17 \leq h \leq 16$, $-8 \leq k \leq 9$, $-27 \leq l \leq 27$
Reflections collected	133447
Independent reflections	9301 [R_{int} = 0.0451, R_{sigma} = 0.0188]
Data/restraints/parameters	9301/58/624
Goodness-of-fit on F^2	1.040
Final R indexes [I>=2σ (I)]	R_1 = 0.0234, wR_2 = 0.0625
Final R indexes [all data]	R_1 = 0.0239, wR_2 = 0.0631
Largest diff. peak/hole / e Å^{-3}	0.39/-0.32
Flack parameter	0.259(5)
CCDC deposition number	**2531520**

Table S11. Fractional Atomic Coordinates (×10^4) and Equivalent Isotropic Displacement Parameters (Å^2×10^3) for 5. U_{eq} is defined as 1/3 of the trace of the orthogonalised U_{IJ} tensor.

Atom	*x*	*y*	*z*	U(eq)
V1	4647.3(3)	9910.7(5)	7081.5(2)	10.66(10)
V2	5290.0(3)	5138.8(5)	2888.9(2)	11.05(10)
S1	4631.5(5)	5459.1(8)	6787.4(3)	17.72(13)
S2	5132.7(6)	8769.0(10)	3793.7(4)	17.77(17)
O13	4784.1(16)	4592(3)	4763.7(9)	25.3(4)
O1	4570.3(14)	11710(2)	7450.4(9)	16.6(4)
O2	3847.2(14)	10389(3)	6323.7(9)	15.0(4)
O4	1585.6(16)	9198(3)	4646.3(10)	26.8(5)
O3	5772.0(14)	10321(2)	6562.7(9)	13.2(4)
O5	8894.2(17)	9806(3)	5676.2(12)	24.1(5)
O6	4735.2(15)	7355(2)	6611.1(8)	17.6(4)
O7	5341.9(14)	3371(3)	2498.0(9)	18.0(4)
O9	6110.2(15)	4597(3)	3626.0(9)	18.3(4)
O14	5274(2)	7659(3)	5372.6(10)	34.0(5)
O15	5151(2)	1327(3)	5227.8(10)	39.9(6)
N1	3407.9(18)	8793(3)	7465.2(10)	15.7(4)
N2	5613.7(17)	8758(3)	7738.7(10)	15.0(4)
O10	8427.2(15)	5621(3)	5286.3(9)	23.6(4)
N4	6533.8(17)	6228(3)	2488.2(10)	15.6(4)
N3	4279.8(17)	6306(3)	2252.9(10)	15.8(4)
O8	4202.1(15)	4775(3)	3439.8(10)	16.7(4)
O11	1092.9(18)	4998(3)	4347.9(12)	24.8(5)
C18	3028(2)	9656(4)	6122.1(13)	13.9(5)
C5	2390(2)	8752(3)	6509.5(13)	15.5(5)
C4	1488(2)	8130(4)	6254.5(14)	18.6(5)
C3	1222(2)	8309(4)	5641.5(14)	20.8(6)
C2	1876(2)	9121(4)	5254.1(13)	18.7(6)
C1	2320(3)	9626(5)	4224.6(14)	24.6(6)
C19	2757(2)	9806(4)	5490.9(14)	17.2(6)
C6	2598(2)	8463(3)	7158.7(13)	16.5(5)
C7	3420(2)	8438(4)	8133.1(12)	19.1(6)
C8	4317(2)	7428(4)	8365.2(12)	20.1(6)
C9	5278(2)	8449(4)	8364.1(12)	19.8(6)
C10	6522(2)	8390(3)	7647.5(12)	16.2(5)
C11	7058(2)	8749(3)	7109.4(12)	14.5(5)
C17	6671(2)	9710(3)	6592.8(13)	13.3(5)
C16	7281(2)	10069(3)	6103.4(15)	15.6(6)
C14	8248(2)	9511(4)	6126.1(13)	17.6(5)
C15	8543(2)	10630(5)	5125.5(14)	27.4(7)
C13	8644(2)	8605(4)	6640.0(14)	20.6(6)
C12	8050(2)	8227(4)	7115.3(13)	19.3(6)
C40	3744(2)	4638(4)	6231.8(16)	24.7(6)

Table S11. Fractional Atomic Coordinates (×10^4) and Equivalent Isotropic Displacement Parameters (Å^2×10^3) for 5. U_{eq} is defined as 1/3 of the trace of the orthogonalised U_{IJ} tensor.

Atom	*x*	*y*	*z*	U(eq)
C39	5693(2)	4496(4)	6491.6(14)	20.5(5)
C37	6949(2)	5262(4)	3820.0(14)	14.8(5)
C38	7239(2)	5068(3)	4448.7(15)	18.0(6)
C21	8124(2)	5722(4)	4677.3(12)	18.2(6)
C20	7680(2)	5297(4)	5710.1(14)	22.0(6)
C22	8775(2)	6554(4)	4290.2(13)	19.1(5)
C23	8495(2)	6758(4)	3679.5(13)	17.5(5)
C24	7586(2)	6170(3)	3428.0(13)	15.3(5)
C25	7350(2)	6528(4)	2788.4(13)	15.8(5)
C26	6475(2)	6733(4)	1832.7(13)	18.2(5)
C27	5557(2)	7774(4)	1655.0(13)	18.9(5)
C28	4618(2)	6724(4)	1635.2(12)	18.6(6)
C29	3384(2)	6663(3)	2358.6(12)	16.3(5)
C30	2866(2)	6243(4)	2905.1(12)	16.1(5)
C36	3287(2)	5298(4)	3408.1(13)	14.5(5)
C35	2695(2)	4881(3)	3907.9(15)	16.0(6)
C33	1714(2)	5355(4)	3894.0(14)	18.6(6)
C34	1483(3)	4206(5)	4901.5(15)	30.3(7)
C32	1290(2)	6263(4)	3389.4(14)	20.9(6)
C31	1864(2)	6704(4)	2912.3(13)	18.6(6)
O12	5330(2)	7908(4)	3187.9(13)	20.1(6)
C41	4406(4)	10586(7)	3559(3)	24.0(9)
C42	6267(4)	9789(6)	4030(3)	28.2(8)
O12A	5227(7)	7604(11)	3442(4)	20.1(6)
S2A	5447.7(19)	9499(4)	3301.2(11)	17.77(17)
C42A	4547(12)	10880(30)	3614(11)	28.2(8)
C41A	6252(11)	10305(18)	3895(7)	24.0(9)

Table S12. Bond Lengths for catalyst **5**.

Atom	Atom	Length/Å	Atom	Atom	Length/Å
V1	O1	1.6096(19)	O11	C33	1.358(4)
V1	O2	1.970(2)	O11	C34	1.431(4)
V1	O3	1.973(2)	C18	C5	1.421(4)
V1	O6	2.2276(19)	C18	C19	1.409(4)
V1	N1	2.107(2)	C5	C4	1.414(4)
V1	N2	2.099(2)	C5	C6	1.443(4)
V2	O7	1.6105(19)	C4	C3	1.371(4)
V2	O9	1.958(2)	C3	C2	1.403(4)
V2	N4	2.119(2)	C2	C19	1.393(4)
V2	N3	2.111(2)	C7	C8	1.521(4)
V2	O8	1.971(2)	C8	C9	1.532(4)
V2	O12	2.235(3)	C10	C11	1.435(4)
V2	O12A	2.255(9)	C11	C17	1.427(4)
S1	O6	1.5215(19)	C11	C12	1.415(4)
S1	C40	1.789(3)	C17	C16	1.408(4)
S1	C39	1.776(3)	C16	C14	1.389(4)
S2	O12	1.509(3)	C14	C13	1.405(4)
S2	C41	1.781(7)	C13	C12	1.373(4)
S2	C42	1.794(5)	C37	C38	1.413(4)
S2	O12A	1.191(9)	C37	C24	1.429(4)
S2	S2A	1.299(3)	C38	C21	1.383(4)
S2	C42A	1.85(2)	C21	C22	1.407(4)
S2	C41A	1.941(15)	C22	C23	1.373(4)
O2	C18	1.313(4)	C23	C24	1.409(4)
O4	C2	1.363(3)	C24	C25	1.439(4)
O4	C1	1.428(4)	C26	C27	1.526(4)
O3	C17	1.315(4)	C27	C28	1.518(4)
O5	C14	1.366(4)	C29	C30	1.447(4)
O5	C15	1.419(4)	C30	C36	1.415(4)
O9	C37	1.309(4)	C30	C31	1.416(4)
N1	C6	1.291(4)	C36	C35	1.420(4)
N1	C7	1.476(3)	C35	C33	1.389(4)
N2	C9	1.473(3)	C33	C32	1.404(4)
N2	C10	1.298(4)	C32	C31	1.371(4)
O10	C21	1.371(3)	O12	O12A	0.622(8)
O10	C20	1.428(4)	O12	S2A	1.262(4)
N4	C25	1.289(4)	C41	S2A	1.766(6)
N4	C26	1.475(3)	C42	S2A	1.911(6)
N3	C28	1.474(3)	O12A	S2A	1.528(9)
N3	C29	1.286(4)	S2A	C42A	1.784(15)
O8	C36	1.314(4)	S2A	C41A	1.770(14)

Table S13. Bond Angles for catalyst **5**.

Atom	Atom	Atom	Angle/°	Atom	Atom	Atom	Angle/°
O1	V1	O2	102.00(10)	C4	C3	C2	118.6(3)
O1	V1	O3	102.46(9)	O4	C2	C3	115.8(3)
O1	V1	O6	177.30(9)	O4	C2	C19	123.4(3)
O1	V1	N1	94.88(10)	C19	C2	C3	120.8(3)
O1	V1	N2	94.62(10)	C2	C19	C18	120.9(3)
O2	V1	O3	84.88(8)	N1	C6	C5	126.9(3)
O2	V1	O6	79.76(8)	N1	C7	C8	113.2(2)
O2	V1	N1	88.90(9)	C7	C8	C9	114.5(2)
O2	V1	N2	163.07(9)	N2	C9	C8	112.8(2)
O3	V1	O6	79.68(8)	N2	C10	C11	127.6(2)
O3	V1	N1	162.45(9)	C17	C11	C10	123.7(3)
O3	V1	N2	88.47(9)	C12	C11	C10	117.6(2)
N1	V1	O6	83.08(8)	C12	C11	C17	118.5(3)
N2	V1	O6	83.75(8)	O3	C17	C11	122.6(3)
N2	V1	N1	92.89(9)	O3	C17	C16	118.4(3)
O7	V2	O9	102.28(10)	C16	C17	C11	118.9(3)
O7	V2	N4	93.70(10)	C14	C16	C17	120.5(3)
O7	V2	N3	93.43(10)	O5	C14	C16	124.6(3)
O7	V2	O8	104.56(10)	O5	C14	C13	114.3(3)
O7	V2	O12	164.35(11)	C16	C14	C13	121.0(3)
O7	V2	O12A	179.5(3)	C12	C13	C14	118.9(3)
O9	V2	N4	88.84(9)	C13	C12	C11	122.1(3)
O9	V2	N3	163.75(10)	O9	C37	C38	118.2(3)
O9	V2	O8	83.88(9)	O9	C37	C24	123.3(3)
O9	V2	O12	87.78(11)	C38	C37	C24	118.4(3)
O9	V2	O12A	77.2(2)	C21	C38	C37	120.7(3)
N4	V2	O12	74.33(10)	O10	C21	C38	123.2(3)
N4	V2	O12A	86.2(2)	O10	C21	C22	115.5(2)
N3	V2	N4	94.33(9)	C38	C21	C22	121.3(3)
N3	V2	O12	77.79(10)	C23	C22	C21	118.2(3)
N3	V2	O12A	87.1(2)	C22	C23	C24	122.7(3)
O8	V2	N4	161.39(9)	C37	C24	C25	123.6(3)
O8	V2	N3	88.20(9)	C23	C24	C37	118.5(3)
O8	V2	O12	88.25(10)	C23	C24	C25	117.9(2)
O8	V2	O12A	75.5(2)	N4	C25	C24	127.3(3)
O12	V2	O12A	15.9(2)	N4	C26	C27	113.1(2)
O6	S1	C40	103.84(13)	C28	C27	C26	114.3(2)
O6	S1	C39	102.99(13)	N3	C28	C27	113.0(2)
C39	S1	C40	98.53(14)	N3	C29	C30	127.2(3)
O12	S2	C41	102.5(2)	C36	C30	C29	123.5(3)
O12	S2	C42	104.9(2)	C36	C30	C31	118.8(3)
O12	S2	C42A	107.3(7)	C31	C30	C29	117.6(3)
O12	S2	C41A	101.4(5)	O8	C36	C30	123.6(3)
C41	S2	C42	101.6(2)	O8	C36	C35	117.7(3)
C41	S2	C42A	9.8(6)	C30	C36	C35	118.7(3)

Table S13. Bond Angles for catalyst **5**.

Atom	Atom	Atom	Angle/°	Atom	Atom	Atom	Angle/°
C41	S2	C41A	88.8(4)	C33	C35	C36	120.4(3)
C42	S2	C42A	92.0(6)	O11	C33	C35	124.4(3)
C42	S2	C41A	14.5(4)	O11	C33	C32	114.7(3)
O12A	S2	O12	23.0(4)	C35	C33	C32	120.9(3)
O12A	S2	C41	119.1(5)	C31	C32	C33	118.9(3)
O12A	S2	C42	113.5(5)	C32	C31	C30	122.2(3)
O12A	S2	S2A	75.6(4)	S2	O12	V2	132.20(18)
O12A	S2	C42A	126.1(8)	O12A	O12	V2	83.9(9)
O12A	S2	C41A	115.3(6)	O12A	O12	S2	48.4(9)
S2A	S2	O12	52.74(17)	O12A	O12	S2A	103.1(9)
S2A	S2	C41	67.9(3)	S2A	O12	V2	171.7(2)
S2A	S2	C42	74.4(2)	S2A	O12	S2	55.07(16)
S2A	S2	C42A	66.5(7)	S2A	C41	S2	42.98(17)
S2A	S2	C41A	62.6(4)	S2	C42	S2A	40.91(14)
C42A	S2	C41A	79.5(7)	S2	O12A	V2	170.9(7)
C18	O2	V1	129.17(18)	S2	O12A	S2A	55.5(4)
C2	O4	C1	116.7(2)	O12	O12A	V2	80.2(9)
C17	O3	V1	131.66(18)	O12	O12A	S2	108.6(11)
C14	O5	C15	118.2(2)	O12	O12A	S2A	53.5(8)
S1	O6	V1	136.83(11)	S2A	O12A	V2	133.6(5)
C37	O9	V2	129.84(19)	S2	S2A	C41	69.1(3)
C6	N1	V1	124.26(19)	S2	S2A	C42	64.72(19)
C6	N1	C7	116.2(2)	S2	S2A	O12A	49.0(4)
C7	N1	V1	119.42(18)	S2	S2A	C42A	71.6(9)
C9	N2	V1	118.88(18)	S2	S2A	C41A	76.8(5)
C10	N2	V1	124.51(18)	O12	S2A	S2	72.2(2)
C10	N2	C9	116.5(2)	O12	S2A	C41	115.3(3)
C21	O10	C20	116.0(2)	O12	S2A	C42	109.9(3)
C25	N4	V2	123.84(19)	O12	S2A	O12A	23.4(3)
C25	N4	C26	116.7(2)	O12	S2A	C42A	124.8(7)
C26	N4	V2	119.45(18)	O12	S2A	C41A	123.6(5)
C28	N3	V2	117.80(18)	C41	S2A	C42	97.7(3)
C29	N3	V2	125.11(19)	C41	S2A	C42A	10.2(6)
C29	N3	C28	117.1(2)	C41	S2A	C41A	94.9(5)
C36	O8	V2	131.87(19)	O12A	S2A	C41	102.8(4)
C33	O11	C34	118.3(3)	O12A	S2A	C42	93.4(4)
O2	C18	C5	123.4(3)	O12A	S2A	C42A	110.4(9)
O2	C18	C19	118.2(3)	O12A	S2A	C41A	108.2(6)
C19	C18	C5	118.3(3)	C42A	S2A	C42	90.2(7)
C18	C5	C6	123.7(3)	C41A	S2A	C42	14.8(4)
C4	C5	C18	118.9(3)	C41A	S2A	C42A	86.0(8)
C4	C5	C6	117.4(3)	S2A	C42A	S2	41.9(4)
C3	C4	C5	122.4(3)	S2A	C41A	S2	40.7(3)

Table S14. Hydrogen Bonds for catalyst **5**.

D	H	A	d(D-H)/Å	d(H-A)/Å	d(D-A)/Å	D-H-A/°
C6	H6	$O7^{1}$	0.95	2.62	3.504(3)	154.1
C8	H8A	S1	0.99	2.98	3.791(3)	139.3
C13	H13	$O7^{2}$	0.95	2.58	3.287(4)	131.2
C40	H40A	O13	0.98	2.62	3.550(4)	159.2
C40	H40C	$O2^{3}$	0.98	2.32	3.290(4)	170.5
C39	H39B	$O3^{3}$	0.98	2.26	3.229(4)	171.6
C25	H25	$O1^{4}$	0.95	2.56	3.436(3)	153.5
C27	H27A	S2A	0.99	2.99	3.825(4)	142.9
C41	H41A	$O7^{5}$	0.98	2.48	3.440(8)	168.2
C42	H42B	$O15^{5}$	0.98	2.42	3.296(6)	148.9
C42A	H42E	$O7^{5}$	0.98	2.61	3.32(3)	129.2
C42A	H42E	$O8^{5}$	0.98	2.19	3.07(2)	149.1
C41A	H41E	$O15^{5}$	0.98	2.53	3.415(15)	149.6
C41A	H41F	$O9^{5}$	0.98	2.42	3.369(14)	163.5
O15	H15D	O13	0.86(3)	1.91(3)	2.753(4)	167(7)
O15	H15E	$O2^{3}$	0.84(3)	2.42(4)	3.123(3)	142(5)
O15	H15E	$O3^{3}$	0.84(3)	2.36(4)	3.084(3)	145(5)

^{1}-1/2+X,1-Y,1/2+Z; ^{2}1/2+X,1-Y,1/2+Z; ^{3}+X,-1+Y,+Z; ^{4}1/2+X,2-Y,-1/2+Z; ^{5}+X,1+Y,+Z

Table S15. Atomic Occupancy for catalyst **5**.

Atom	*Occupancy*	Atom	*Occupancy*	Atom	*Occupancy*
S2	0.7335(15)	O12	0.7335(15)	C41	0.7335(15)
H41A	0.7335(15)	H41B	0.7335(15)	H41C	0.7335(15)
C42	0.7335(15)	H42A	0.7335(15)	H42B	0.7335(15)
H42C	0.7335(15)	O12A	0.2665(15)	S2A	0.2665(15)
C42A	0.2665(15)	H42D	0.2665(15)	H42E	0.2665(15)
H42F	0.2665(15)	C41A	0.2665(15)	H41D	0.2665(15)
H41E	0.2665(15)	H41F	0.2665(15)		

References

(1) Tsumaki, T. Nebenvalenzringverbindungen. IV. Über Einige Innerkomplexe Kobaltsalze Der Oxyaldimine. *Bull. Chem. Soc. Jpn.* **1938**, *13* (2), 252–260.

(2) Helaly, A. A.; Babgi, B. A.; Kobayashi, Y.; Rai, R. K.; Ali, E. M. M.; Kalantan, A. A.; Hassan, W. M. I.; Hussien, M. A.; Ismail, M. M. I. Salen-Type Copper(II) Complexes: Synthesis, Characterization, Computational Studies, Molecular Docking, Anticancer Potential, and Pharmacokinetic Prediction. *ChemistryOpen* **2025**, *14* (9), e202500061. DOI: 10.1002/open.202500061.

(3) Liu, Q.; Ding, M.; Lin, Y.; Xing, Y. Trans-(±)-N,N′-Bis(Salicylidene)-1,2-Cyclohexanediamine. *Acta Crystallogr. C* **1997**, *53* (11), 1671–1673. DOI: 10.1107/S0108270197008640.

(4) Yu, C.; Zhang, Z.; Liu, L.; Li, H.; He, Y.; Lü, X.; Wong, W. K.; Jones, R. A. PMMA-Supported Hybrid Materials Doped with Highly near-Infrared (NIR) Luminescent Complexes [Zn(L1)(Py)Ln(L2)3] (Ln = Nd, Yb or Er). *New J. Chem.* **2015**, *39* (5), 3698–3707. DOI: 10.1039/c4nj02373k.

(5) Atakol, O.; Nazir, H.; Arici, C.; Durmus, S.; Svoboda, I.; Fuess, H. Some New Ni-Zn Heterodinuclear Complexes: Square-Pyramidal(II) Coordination. *Inorg. Chim. Acta* **2002**, *342*, 295–300. DOI: 10.1016/S0020-1693(02)01163-5.

(6) Elamin, N. Y.; Elamin, M. R.; Abdalla, S.; Khalil, M. A.; Helaly, A. A.; Babgi, B. A.; Hussien, M. A. Bioengineered Nanocellulose–Schiff Base Sensor for Selective Fluorometric Detection of Cd2+ Ions in Aqueous Media. *Int. J. Biol. Macromol.* **2025**, *328*, 147594. DOI: 10.1016/j.ijbiomac.2025.147594.

(7) Maclachlan, M. J.; Park, M. K.; Thompson, L. K. Coordination Compounds of Schiff-Base Ligands Derived from Diaminomaleonitrile (DMN): Mononuclear, Dinuclear, and Macrocyclic Derivatives. *Inorg. Chem.* **1996**, *35*, 5492–5499.

(8) Selvarani, V.; Annaraj, B.; Neelakantan, M. A.; Sundaramoorthy, S.; Velmurugan, D. Synthesis, Characterization and Crystal Structures of Copper(II) and Nickel(II) Complexes of Propargyl Arm Containing N2O2 Ligands: Antimicrobial Activity and DNA Binding. *Polyhedron* **2013**, *54*, 74–83. DOI: 10.1016/j.poly.2013.02.030.

(9) Riley, P. E.; Pecoraro, V. L.; Carrano, C. J.; Bonadies, J. A.; Raymond, K. N. Contribution from the X-Ray Crystallographic Characterization of a Stepwise, Metal-Assisted Oxidative Decarboxylation: Vanadium Complexes of Ethylenebis[(o-Hydroxyphenyl)Glycine] and Derivatives. *Inorg. Chem* **1986**, *25*, 154–160.

(10) Mathew, M.; Carty, A. J.; Palenik, G. J. An Unusual Complex Containing Bridging Vanadyl Groups. The Crystal Structure of N,N'-Propylenebis(Salicylaldiminato)Oxovanadium(IV). *J. Am. Chem. Soc.* **1970**, *92* (10), 3197–3198.

(11) Zang, T. T.; Cao, J. P.; Du, Z. Y.; Mei, H.; Xu, Y. Two New Schiff-Base Modified Vanadium Complexes with Third-Order NLO Properties. *J. Coord. Chem.* **2020**, *73* (17–19), 2763–2772. DOI: 10.1080/00958972.2020.1827144.

(12) Kaur, A.; Bhardwaj, N.; Kaur, A.; Abida, K.; Nagaraja, T. P.; Ali, A.; Prakash, R. Proton Nuclear Magnetic Resonance-Based Method for the Quantification of Epoxidized Methyl Oleate. *J. Am. Oil Chem. Soc.* **2021**, *98* (2), 139–147. DOI: 10.1002/aocs.12439.

(13) Hiatt, R.; Smythe, R. J.; Mccoleman, C. The Reaction of Hydroperoxides with Triphenylphosphine. *Can. J. Chem.* **1971**, *49*, 1707.

(14) Frisch, M. J. ; Trucks, G. W. ; Schlegel, H. B. ; Scuseria, G. E. ; Robb, M. A. ; Cheeseman, J. R. ; Scalmani, G. ; Barone, V. ; Petersson, G. A. ; Nakatsuji, H. ; Li, X. ; Caricato, M.; Marenich, A. V. ; Bloino, J. ; Janesko, B. G. ; Gomperts, R. ; Mennucci, B. ; Hratchian, H. P. ; Ortiz, J. V. ; Izmaylov, A. F. ; Sonnenberg, J. L. ; Williams-Young, D. ; Ding, F. ; Lipparini, F. ; Egidi, F. ; Goings, J. ; Peng, B. ; Petrone, A.; Henderson, T. ; Ranasinghe, D. ; Zakrzewski, V. G. ; Gao, J. ; Rega, N. ; Zheng, G. ; Liang, W. ; Hada, M. ; Ehara, M. ; Toyota, K. ; Fukuda, R. ; Hasegawa, J. ; Ishida, M. ; Nakajima, T.; Honda, Y. ; Kitao, O. ; Nakai, H. ; Vreven, T. ; Throssell, K. ; Montgomery, J. A. , Jr. ; Peralta, J. E. ; Ogliaro, F. ; Bearpark, M. J. ; Heyd, J. J. ; Brothers, E. N. ; Kudin, K. N. ; Staroverov, V. N. ; Keith, T. A. ; Kobayashi, R. ; Normand, J.; Raghavachari, K. ; Rendell, A. P. ; Burant, J. C. ; Iyengar, S. S. ; Tomasi, J. ; Cossi, M. ; Millam, J. M. ; Klene, M. ; Adamo, C. ; Cammi, R. ; Ochterski, J. W. ; Martin, R. L.; Morokuma, K. ; Farkas, O. ; Foresman, J. B. ;; Fox, D. J. Gaussian 09, Revision A.02. Gaussian, Inc., Wallingford CT 2016. https://gaussian.com/g09citation/.

(15) Becke, A. D. Density-Functional Thermochemistry. III. The Role of Exact Exchange. *J. Chem. Phys.* **1993**, *98* (7), 5648–5652. DOI: 10.1063/1.464913.

(16) Andrae, D.; Hiuflermann, U.; Doig, M.; Stoll, H.; Preull, H. Theorefica Chimica Acta Energy-Adjusted Ab Initio Pseudopotentials for the Second and Third Row Transition Elements: Molecular Test for M2 (M = Ag, Au) and MH (M = Ru, Os). *Theor. Chim. Acta* **1991**, *78*, 247–266.

(17) Grimme, S.; Antony, J.; Ehrlich, S.; Krieg, H. A Consistent and Accurate Ab Initio Parametrization of Density Functional Dispersion Correction (DFT-D) for the 94 Elements H-Pu. *J. Chem. Phys.* **2010**, *132* (15), 154104. DOI: 10.1063/1.3382344.

(18) Reed, A. E.; Curtiss, L. A.; Weinhold, F. Intermolecular Interactions from a Natural Bond Orbital, Donor-Acceptor Viewpoint. *Chem. Rev.* **1988**, *88* (6), 899–926.

(19) Sheldrick, G. M. SHELXT - Integrated Space-Group and Crystal-Structure Determination. *Acta Crystallogr. A* **2015**, *71* (1), 3–8. DOI: 10.1107/S2053273314026370.

(20) Sheldrick, G. M. A Short History of SHELX. *Acta Crystallogr. A* **2008**, *64* (1), 112–122. DOI: 10.1107/S0108767307043930.

(21) Dolomanov, O. V.; Bourhis, L. J.; Gildea, R. J.; Howard, J. A. K.; Puschmann, H. OLEX2: A Complete Structure Solution, Refinement and Analysis Program. *J. Appl. Crystallogr.* **2009**, *42* (2), 339–341. DOI: 10.1107/S0021889808042726.
